# Inositol triphosphate-triggered calcium release blocks lipid exchange at endoplasmic reticulum-Golgi contact sites

**DOI:** 10.1038/s41467-021-22882-x

**Published:** 2021-05-11

**Authors:** Mouhannad Malek, Anna M. Wawrzyniak, Peter Koch, Christian Lüchtenborg, Manuel Hessenberger, Timo Sachsenheimer, Wonyul Jang, Britta Brügger, Volker Haucke

**Affiliations:** 1 Leibniz-Forschungsinstitut für Molekulare Pharmakologie (FMP), Berlin, Germany; 2 Heidelberg University Biochemistry Center (BZH), Heidelberg University, Heidelberg, Germany; 3 Faculty of Biology, Chemistry and Pharmacy, Freie Universität Berlin, Berlin, Germany

**Keywords:** Lipidomics, Lipid signalling, Endocytosis

## Abstract

Vesicular traffic and membrane contact sites between organelles enable the exchange of proteins, lipids, and metabolites. Recruitment of tethers to contact sites between the endoplasmic reticulum (ER) and the plasma membrane is often triggered by calcium. Here we reveal a function for calcium in the repression of cholesterol export at membrane contact sites between the ER and the Golgi complex. We show that calcium efflux from ER stores induced by inositol-triphosphate [IP_3_] accumulation upon loss of the inositol 5-phosphatase INPP5A or receptor signaling triggers depletion of cholesterol and associated Gb3 from the cell surface, resulting in a blockade of clathrin-independent endocytosis (CIE) of Shiga toxin. This phenotype is caused by the calcium-induced dissociation of oxysterol binding protein (OSBP) from the Golgi complex and from VAP-containing membrane contact sites. Our findings reveal a crucial function for INPP5A-mediated IP_3_ hydrolysis in the control of lipid exchange at membrane contact sites.

## Abstract (web summary)

The interplay between non-vesicular lipid transport, calcium signaling, and membrane dynamics is unclear. Here, the authors report a function for inositol triphosphate hydrolysis by the inositol 5-phosphatase INPP5A in controlling lipid exchange at interorganelle membrane contact sites between the endoplasmic reticulum and Golgi.

## Introduction

Cellular membrane homeostasis and the exchange of material between compartments can occur by vesicular traffic along the endocytic and secretory pathways^1,2^, and by membrane contact sites (MCS), i.e., areas where organelles are in close apposition^3–7^. For example, the endoplasmic reticulum (ER) has been found to form MCS with essentially all other organelles including the plasma membrane, mitochondria, endosomes, lysosomes, and the trans-Golgi network (TGN) to facilitate calcium homeostasis and signaling^8,9^ and the exchange of lipids^5,7,10^. A major physiological role of MCS is non-vesicular lipid exchange by lipid-transport proteins, such as the members of the oxysterol binding protein (OSBP) family. The founding member OSBP has been shown to exchange cholesterol, a major constitutent of the plasma membrane, and phosphatidylinositol 4-phosphate [PI(4)P], a lipid enriched in the Golgi complex, at MCS between the ER and the TGN formed by ER-localized VAP-A/B proteins that act as membrane tethers^11–13^. Inhibition of OSBP function causes the re-routing of cholesterol to ER-derived lipid droplets and the concomitant depletion of cholesterol from the Golgi complex and, partially, from the plasma membrane^11^.

Cholesterol tightly associates with glycosphingolipids and proteins in cell membranes^14,15^. Hence, loss of cholesterol is expected to affect a multitude of cellular functions^16–20^. These range from the formation of glycosphingolipid-dependent signaling and trafficking platforms^17,18,21^ to the clathrin-independent endocytosis (CIE) of cell adhesion molecules^22^ and bacterial toxins, e.g., Shiga toxin^23–25^. Cellular uptake of Shiga toxin is initiated by binding to the cholesterol-associated^25^ complex glycosphingolipid globotriosylceramide (Gb3) followed by cholesterol-dependent membrane reorganization^24^, that results in the formation and fission of endocytic membrane vesicles^26^. Similar pathways of CIE control the surface levels and activity of cell signaling receptors^27,28^, including receptors (e.g., G protein-coupled receptors) linked to phospholipase C (PLC). This enzyme hydrolyzes plasma membrane phosphatidylinositol 4,5-bisphosphate [PI(4,5)P_2_] to generate the soluble messenger inositol 1,4,5-triphosphate [IP_3_], which triggers an increase in cytoplasmic calcium levels^29,30^ that, in turn, may affect MCS^5,8^. Intracellular IP_3_ levels are controlled in part by inositol phosphatases such as the ubiquitously expressed plasma membrane-associated^31^ inositol 5-phosphatase INPP5A, which specifically hydrolyzes IP_3_ (and IP_4_) to repress calcium signaling^32^ and is downregulated in cancer and spinocerebellar ataxia type 17^33–37^. These findings suggest a close, albeit poorly understood, interplay between non-vesicular lipid transport via membrane contact sites, calcium signaling, and the cholesterol-dependent and glycosphingolipid-dependent formation of endocytic vesicles during CIE.

Using an siRNA-based screening approach paired with functional imaging, we found that IP_3_-triggered calcium efflux from the ER in the absence of INPP5A blocks Shiga toxin endocytosis as a result of impaired cholesterol transport to the Golgi complex, and the concomitant partial depletion of cholesterol and the cholesterol-associated complex glycosphingolipid Gb3 from the plasma membrane. We further demonstrate that this phenotype is a consequence of the calcium-induced dissociation of OSBP from the TGN and from VAP-containing MCS. The inhibitory role of calcium with respect to OSBP/ VAP-mediated MCS formation is, thus, distinct from the established function of calcium in facilitating STIM1-ORAI channel formation^9,29^, and the recruitment of ER-localized E-Syts to ER-plasma membrane contact sites^5,7,38^.

These results have important implications for our understanding of the interplay between calcium signaling and cellular lipid homeostasis and bear implications for diseases, such as cancer and ataxia associated with altered INPP5A levels or function.

## Results

### INPP5A is required for Gb3-mediated Shiga toxin cell entry

**Fig. 1:**
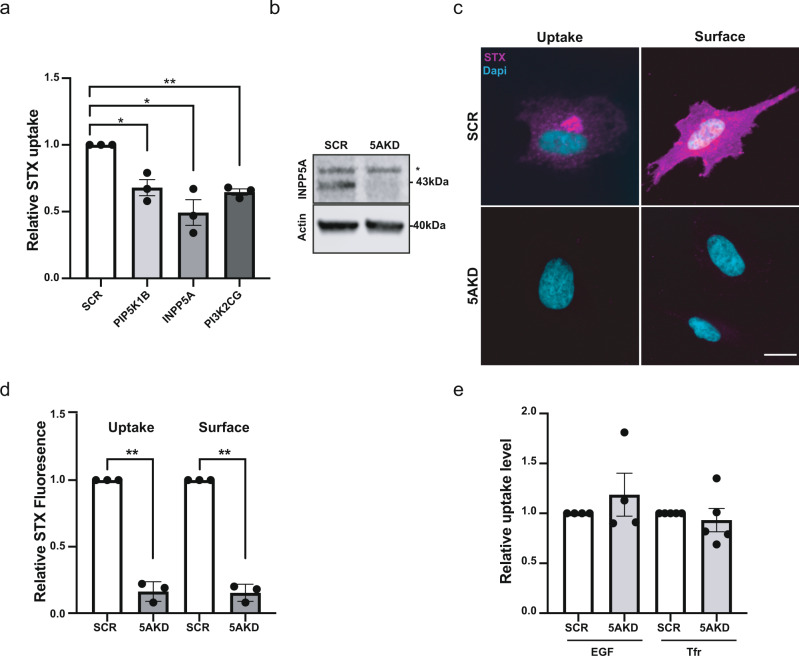
INPP5A loss impairs CIE of Shiga toxin. **a** Relative Shiga toxin (STX; 5 μg/ml) uptake in HeLa cells treated with siRNAs against the indicated proteins. STX uptake into SCR-siRNA-treated control cells (*n* = 50 cells, set to 1) or into cells depleted of INPP5A (*n* = 47 cells), PIP5K1B (*n* = 86 cells), or PI3KC2γ (PI3K2CG, *n* = 47 cells). One sample *t*-test with Benjamini–Hochberg correction: PIP5K1B: *p* = 0.0343, *t* = 5.261, df = 2. INPP5A: *p* = 0.0343, *t* = 5.279, df = 2. PI3K2CG: *p* = 0.0138, *t* = 14.7, df = 2. Data represent mean ± SEM, *n* = 3 independent experiments. **b** Representative immunoblot from three independent experiments for INPP5A and β-actin of HeLa cells treated with control (SCR) or INPP5A siRNA (5AKD). Asterisk denotes non-specific background band decorated by INPP5A antibodies. **c** Representative confocal images of HeLa cells treated with siRNA control (SCR) or INPP5A siRNA (5AKD) and incubated with labeled STX (magenta) for 15 min at 37 °C (uptake) or kept at 4 °C for 45 min (surface binding). Blue, DAPI-stained nuclei. Scale bar, 25 μm. **d** Quantification of cells similar to those shown in **c**. Data for SCR-control siRNA-treated cells were set to 1. One sample *t*-test for each pair. Uptake: *p* = 0.0026, *t* = 19.66, df = 2. Surface: *p* = 0.0019, *t* = 22.81, df = 2. Data represent mean ± SEM from three independent experiments, [number of cells for uptake: (n1) SCR: 329, INPP5A: 243, (n2) SCR: 341, INPP5A: 261 (n3) SCR: 295, INPP5A: 344, Surface: (n1) SCR: 54, INPP5A: 104, (n2) SCR: 340, INPP5A: 183 (n3) SCR: 243, INPP5A: 205]. **e** Uptake of EGF or transferrin in HeLa cells treated with control (SCR, set to 1) or INPP5A siRNA (5AKD). One sample *t*-test, EGF: *p* = 0.4458, *t* = 0.8754, df = 3. Trf: *p* = 0.5912, *t* = 0.5830, df = 3. Data are mean ± SEM from *n* = 4 (EGF uptake) or 5 (Tfr uptake) independent experiments. Numerical source data and unprocessed blots are reported in the Source Data file.

Given the pivotal roles of inositol lipids in the regulation of membrane traffic^1,39^ we screened an siRNA smart pool library encoding all known human inositol kinases and phosphatases for a possible function in the internalization of Shiga toxin, a bacterial toxin known to enter cells via a glycosphingolipid-mediated clathrin-independent endocytosis (CIE) pathway. Consistent with the established function of phosphatidylinositol-bisphosphates in endocytosis^1,27,40^, we found CIE of Shiga toxin to be strongly reduced upon cellular depletion of phosphatidylinositol (PI) 4-phosphate 5-kinase type IB (PIPKIB) or PI 3-kinase C2γ (PI3KC2G), i.e., enzymes that synthesize plasma membrane PI(4,5)P_2_ or phosphatidylinositol (3,4)-bisphosphate [PI(3,4)P_2_]^40,41^, respectively (Fig. 1a). Our attention, however, was caught by the adverse effects of knockdown of the inositol 5-phosphatase INPP5A, an enzyme known to predominantly hydrolyze non-membrane bound soluble inositol phosphates such as inositol 1,4,5-triphosphate [IP_3_]^34,36,42^, the product of PI(4,5)P_2_ cleavage by phospholipase C downstream of receptor signaling^32^. Hence, we explored the molecular pathway and mechanism underlying the putative role of INPP5A in CIE.

Shiga toxin endocytosis is known to be initiated by the tight association of its oligomeric B subunit with Gb3 glycosphingolipids on the cell surface^25,26,28^. Defective CIE of Shiga toxin in INPP5A-depleted cells therefore could either reflect a requirement for INPP5A in the internalization step of CIE, or in the regulation of Gb3 levels at the cell surface. Impaired CIE of Shiga toxin upon effective knockdown of INPP5A (Fig. 1b) was paralleled by a proportional decrease in binding of Shiga toxin to the surface of INPP5A-depleted HeLa cells (Fig. 1c, d), suggesting that Gb3 is depleted from the plasma membrane of cells lacking INPP5A. A requirement for INPP5A in the Gb3-dependent uptake of Shiga toxin was further confirmed in *INPP5A* knockout (KO) HAP1 cells generated by CRISPR/Cas9 (Supplementary Fig. 1a). In contrast, CME of transferrin or internalization of EGF proceeded unperturbed in INPP5A-depleted cells (Fig. 1e and Supplementary Fig. 1b). No alterations in the amounts of transferrin or EGF receptors at the plasma membrane, or in the levels of surface glycoproteins detected by wheat germ agglutinin were detected upon loss of INPP5A (Supplementary Fig. 1c, d). These data indicate that INPP5A controls the cell surface levels of Gb3, and thereby, CIE of bacterial Shiga toxin.

### Loss of INNP5A reduces cell surface cholesterol and Gb3

**Fig. 2:**
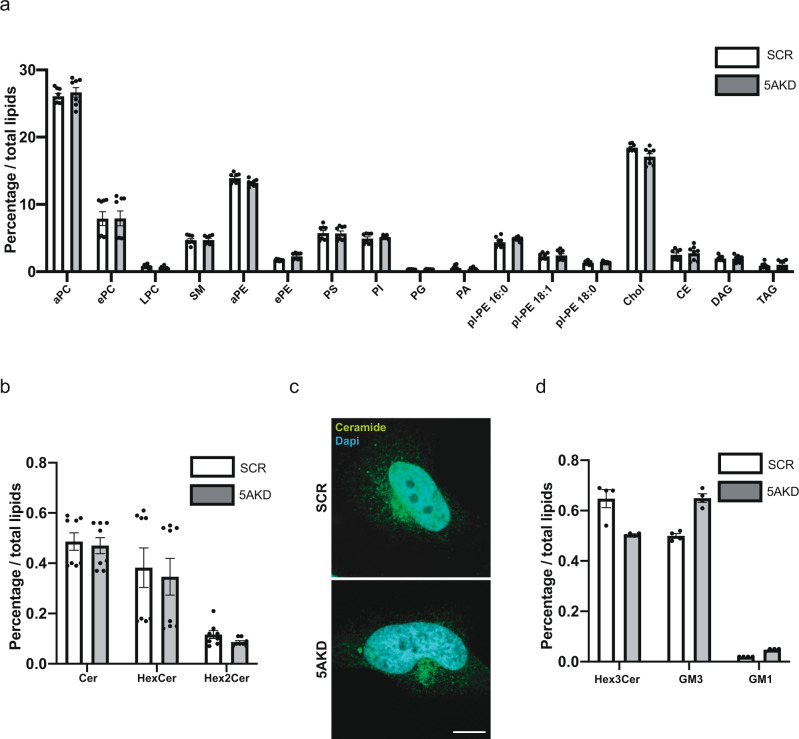
Total cellular lipid levels in absence of INPP5A. **a**, **b**, **d** Lipidomic analyses. SCR, control (SCR), 5AKD, INPP5A-depleted HeLa cells. Ceramide (Cer), cholesterol (Chol), cholesteryl ester (CE), diacylglycerol (DAG), dihexosylceramide (Hex2Cer), ganglioside GM1 (GM1), ganglioside GM3 (GM3), globoside Gb3 /trihexosylceramide, (Hex3Cer), hexosylceramide (HexCer), lyso-PC (LPC), phosphatidic acid (PA), phosphatidylcholine (PC), phosphatidylethanolamine (PE), phosphatidylglycerol (PG), phosphatidyinositol (PI), phosphatidylserine (PS), plasmalogen (plasmenyl) ethanolamine (pl-PE), sphingomyelin (SM), triacylglycerol (TAG). a = acyl-linked glycerophospholipids, e = ether-linked (plasmanyl) or containing one odd-chain fatty acyl. Two-sided *t*-test corrected for multiple comparisons using the Holm-Sidak method: aPC: *p* = 0.49, *t* = 0.69, df = 14. ePC: *p* = 0.98, *t* = 0.02, df = 14. LPC: *p* = 0.076, *t* = 1.91, df = 14. SM: *p* = 0.90, *t* = 0.1252, df = 14.aPE: *p* = 0.033, *t* = 2.35, df = 14. ePE: *p* = 0.005, *t* = 3.239, df = 14 aPS: *p* = 0.93, *t* = 0.08, df = 14. pl-PE 16:0: *p* = 0.077, *t* = 1.90, df = 14. pl-PE 18:1: *p* = 0.700, *t* = 2.29, df = 14. pl-PE 18:0: *p* = 0.49, *t* = 0.6964, df = 14. Chol: *p* = 0.013, *t* = 2.814, df = 14. CE: *p* = 0.644, *t* = 0.47, df = 14. DAG: *p* = 0.92, *t* = 0.1001, df = 14. TAG: *p* = 0.56, *t* = 0.58, df = 14. Cer: *p* = 0.734, *t* = 0.346, df = 14. HexCer: *p* = 0.74, *t* = 0.33 df = 14. Hex2Cer: p = 0.093, t = 1.79, df = 14. Hex3Cer: *p* = 0.007, *t* = 3.955, df = 6. GM3: *p* = 0.0002, *t* = 7.83, df = 6. GM1: *p* = 0.000034, *t* = 11, df = 6. **c** Confocal images of control (SCR) or INPP5A (5AKD)-depleted HeLa cells stained for ceramide (green). Blue, DAPI. Scale bar, 10 μm. Data are mean ± SEM from 8 (**a**, **b**), 3 (**c**), or 4 (**d**) independent experiments. For numerical source data see Source Data file.

Reduced cell surface levels of Gb3 in INPP5A-depleted cells could arise either from (i) defects in complex glycosphingolipid biosynthesis, or (ii) be caused indirectly as a consequence of impaired lipid transport, e.g., by defective transport of Gb3 and/or associated lipids such as cholesterol^25^ from the Golgi complex, i.e., their site of synthesis, to the plasma membrane. To examine whether INPP5A loss impacts on total cellular lipid composition, we quantitatively analyzed lipids from total extracts of control and INPP5A-depleted HeLa cells by nano‐electrospray ionization tandem mass spectrometry as described^43,44^. No major alterations were detected in the amounts or relative fractions of glycerophospholipids (e.g., phosphatidylcholine, phosphatidylethanolamine, phosphatidylserine, phosphatidic acid, phosphatidylinositol, phosphatidylglycerol) and neutral lipids (diacylglycerol and triacylglycerol) (Supplementary Data File 1). The total cellular amounts of cholesterol or sphingomyelin were also largely unaltered (≤10%, i.e., the error of analysis) (Fig. 2a). We also failed to detect any changes in the amounts of ceramide, the main substrate for sphingolipid synthesis, in the levels of simple glycosphingolipids such as monohexosylceramides or di-hexosylceramides (e.g., lactosylceramide) (Fig. 2b). Unaltered levels and distribution of ceramide, the common precursor for all glycosphingolipids^45^ was confirmed by confocal image analysis of HeLaM cells stained with antibodies against ceramide (Fig. 2c). The total content of the complex glycosphingolipids GM3 and GM1 was moderately increased, while that of tri-hexosylceramides including Gb3 was marginally decreased (to about 77% of controls) in INPP5A-depleted cells (Fig. 2d). Hence, loss of INPP5A does not alter the total cellular glycerophospholipid to glycosphingolipid ratio, and leads to minor changes in complex glycosphingolipid composition (Fig. 2 and Supplementary Data File 1). These results make it unlikely that INPP5A loss inhibits CIE of Shiga toxin by blocking lipid biosynthesis.

We therefore followed the alternative hypothesis that the depletion of Gb3 from the cell surface might reflect defects in its transport to the plasma membrane. Earlier work had revealed a close physical association of complex glycosphingolipids including Gb3 with cholesterol including the formation of microdomains at the levels of the Golgi complex^46^ and the plasma membrane^17,18,21,25,47^. We therefore hypothesized that the reduced surface levels of Gb3 might be a consequence of defective cholesterol export from the ER in the absence of INPP5A.

**Fig. 3:**
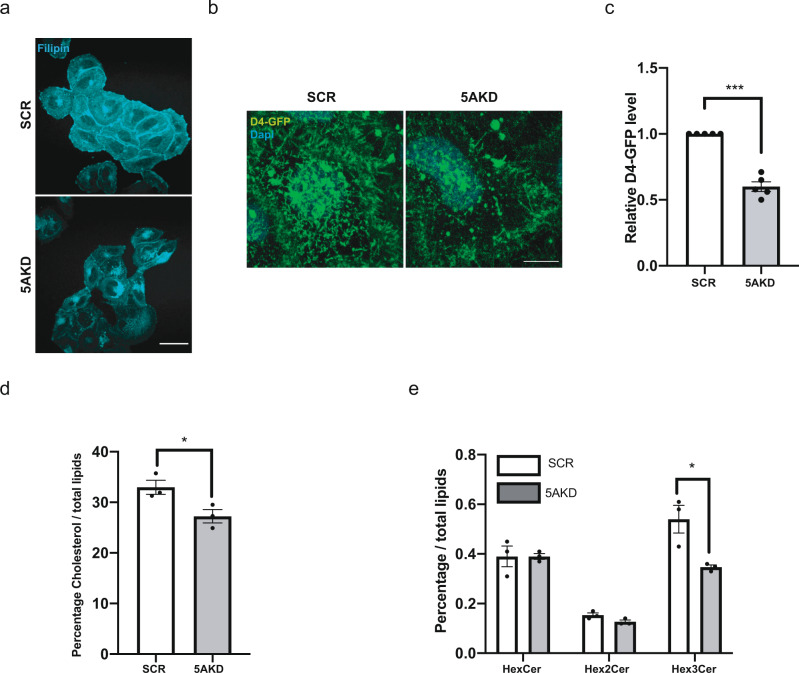
Cholesterol is partially depleted from the cell surface in the absence of INPP5A. **a** Representative epifluorescent images from four independent experiments of HeLa cells grown in serum-containing media treated with control (SCR) or INPP5A siRNA (5AKD) and incubated for 45 min post-fixation with 0.05 mg/ml Filipin (cyan). Scale bar, 25 μm. **b** Representative *z*-projections from confocal images of HeLa cells grown in serum-containing media treated with control (SCR) or INPP5A siRNA (5AKD) and stained for plasma membrane cholesterol using recombinant D4-GFP (green). Blue, DAPI-stained nuclei. Scale bar, 10 μm. **c** Relative D4-GFP level as a measure for plasma membrane cholesterol in HeLa cells treated with control (SCR) or INPP5A siRNA (5AKD). One sample *t*-test 5AKD: *p* = 0.0004, *t* = 10.97, df = 4. Data represent mean ± SEM from five independent experiments (**d**, **e**) Lipidomic analysis of plasma membrane-enriched fractions from control (scrambled siRNA, SCR) and INPP5A-depleted (5AKD) HeLa cells grown in serum-containing media. Depicted are free cholesterol, trihexosylceramide (Hex3Cer, i.e., Gb3), dihexosylceramide (Hex2Cer), and hexosylceramide (HexCer). Two sided *t*-test: Cholesterol: *p* = 0.0411, *t* = 2.971, df = 4. Two sided *t*-test corrected for multiple comparisons using the Holm-Sidak method: HexCer: *p* > 0.999, *t* = 0, df = 4. Hex2Cer: *p* = 0.073, *t* = 2.412, df = 4. Hex2Cer:*p* = 0.027, *t* = 3.43, df = 4. Data represent mean ± SEM from three independent experiments. Numerical source data are reported in the Source Data file.

**Fig. 4:**
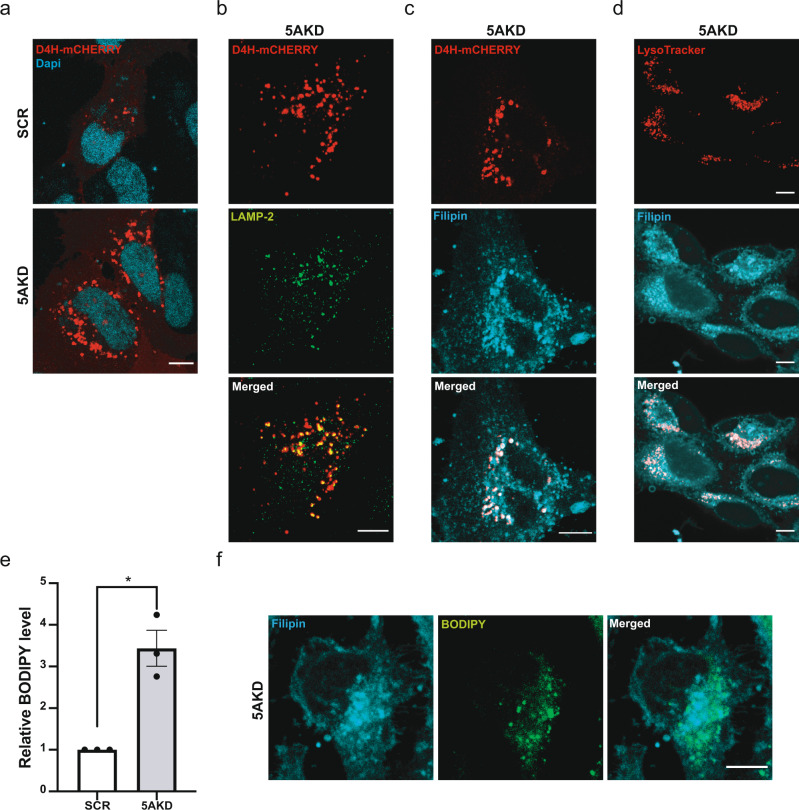
In the absence of INPP5A cholesterol is directed towards lysosomes and lipid droplets. **a** Representative images from three independent experiments of HeLa cells treated with control (SCR) or INPP5A siRNA (5AKD) and transfected with a plasmid encoding D4H-mCherry (red) to detect intracellular cholesterol, Blue, DAPI-stained nuclei. Scale bar, 10 μm. **b** Representative images from three independent experiments of HeLa cells treated with INPP5A siRNA (5AKD), transfected with a plasmid encoding D4H-mCherry (red) to detect intracellular cholesterol, and stained with anti-LAMP-2(green) antibodies to label lysosomes. Scale bar, 10 μm. **c** Representative images from three independent experiments of HeLa cells treated with INPP5A siRNA (5AKD), transfected with a plasmid encoding D4H-mCherry (red) to detect intracellular cholesterol, and incubated for 45 min post-fixation with 0.05 mg/ml Filipin (cyan). Scale bar, 10 μm. **d** Representative images from two independent experiments of HeLa cells treated with INPP5A siRNA (5AKD), incubated with Lysotracker (red, 1:1000 dilution) for 60 min before fixation to stain lysosomes, and incubated for 45 min post-fixation with 0.05 mg/ml Filipin (cyan). Scale bar, 10 μm. **e** Relative BODIPY 493/503 (4,4-Difluoro-1,3,5,7,8-Pentamethyl-4-Bora-3a,4a-Diaza-s-Indacene) levels incubated for 15 min with 2 μM as a measure for lipid droplets in HeLa cells treated with control (SCR) or INPP5A siRNA (5AKD). One sample *t*-test: 5AKD: *p* = 0.03, *t* = 5.642, df = 2. **f** Representative images from three independent experiments of HeLa cells treated with INPP5A siRNA (5AKD) and incubated for 15 min with 2 μM of BODIPY (green) before fixation and incubated for 45 min post-fixation with 0.05 mg/ml Filipin (cyan) Scale bar, 10 μm. All data represent mean ± SEM from three independent experiments. Numerical source data are reported in the Source Data file.

Visualization of the overall distribution of cholesterol by Filipin suggested that cholesterol is partially redistribruted from the cell surface to intracellular organelles in INPP5A-depleted cells (Fig. 3a). Moreover, specific labeling of the free pool of plasma membrane cholesterol with the recombinant purified GFP-tagged domain 4 (D4) of Perfringolysin O (PFO, theta toxin), a high-affinity cholesterol-binding protein with a non-linear response^48^, revealed reduced cholesterol levels in the exoplasmic plasma membrane leaflet of INPP5A-depleted cells compared to SCR siRNA-treated controls (Fig. 3b, c). Quantitative mass spectrometric analysis of lipids extracted from plasma membrane-enriched fractions confirmed the reduction of total cholesterol in INPP5A-depleted cells compared to control cells (Fig. 3d), although in this case the reduction appeared less pronounced compared to the cholesterol pool detected by GFP-D4. The change in plasma membrane cholesterol was paralleled by a near two-fold reduction in plasma membrane trihexosylceramide, i.e., Gb3, levels in INPP5A knockdown cells, whereas monohexosylceramides and dihexosylceramides were unaffected by INPP5A loss (Fig. 3e). These data show that loss of INPP5A leads to a partial depletion of cholesterol and, more pronouncedly, Gb3 from the plasma membrane, possibly as a result of defective lipid transport. To further examine this possibility, we monitored the intracellular distribution of cholesterol in INPP5A-depleted cells using two complementary probes: (1) Filipin, a cholesterol binding dye that detects cholesterol in intracellular membranes including lipid droplets enriched in cholesterol esters^49^, and (2) a cytoplasmically expressed chimera of mCherry with domain 4 of Perfringolysin O theta toxin (D4H)^48^. INPP5A-depleted cells displayed an accumulation of intracellular cholesterol puncta detected by cytosolic expression of D4H-mCherry (Fig. 4a), that largely overlapped with cholesterol staining by Filipin (Fig. 4c). Colocalization analysis using organellar markers showed that D4H-mCherry or Filipin-labeled puncta partially overlapped with lysosomes immunopositive for LAMP2 (i.e., in case of D4H-mCherry) or labeled live by lysotracker (Fig. 4b, d and Supplementary Fig. 2a). INPP5A-depleted cells also showed an increase in the number of BODIPY (493/503)-marked lipid droplets (Fig. 4e and Supplementary Fig. 2b), that partially overlapped with Filipin at the confocal microscopy level (Fig. 4f). These data suggest that in the absence of INPP5A cholesterol export to the cell surface is compromised. Instead, cholesterol is re-routed to lysosomes and, to lipid droplets that emanate from ER membranes. Consistently, we observed reduced levels of the latent transcription factor SREBP-1 (Supplementary Fig. 4c, d), a sensor of ER cholesterol content whose expression is known to be positively autoregulated via binding to its own promoter^50–53^.

### INPP5A regulates OSBP-mediated cholesterol export

**Fig. 5:**
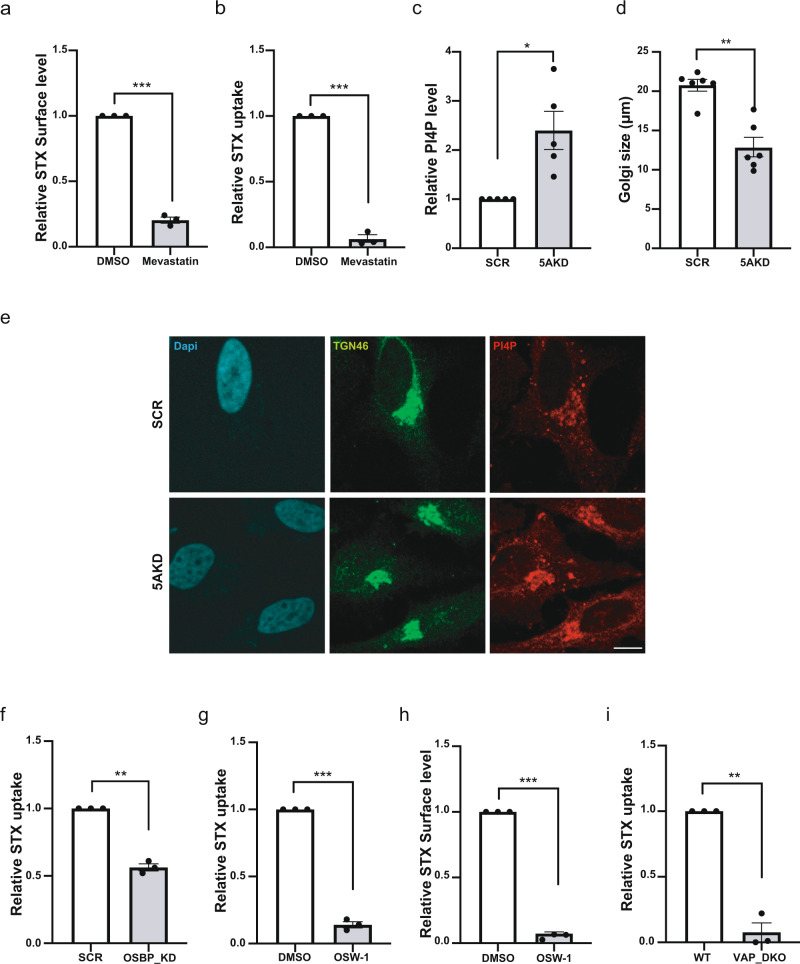
Impaired cholesterol/ PI(4)P lipid exchange at ER/ Golgi membrane contact sites blocks CIE of Shiga toxin. **a** Relative STX surface level in HeLa cells treated with DMSO (set to 1) or depleted of cholesterol by Mevastatin (250 nM) for 24 h in presence of serum. One sample *t*-test: *p* = 0.0009, *t* = 34.14, df =2. **b** Relative STX uptake level in HeLa cells treated with DMSO (set to 1) or depleted of cholesterol by Mevastatin (250 nM) for 24 h in presence of serum. One sample *t*-test: *p* = 0.0009, *t* = 32.89, df = 2. **c** Relative phosphatidylinositol 4-phosphate [PI(4)P] levels in HeLa cells treated with control (SCR, set to 1) or INPP5A siRNA (5AKD). One sample *t*-test: *p* = 0.0229, *t* = 3.594, df = 4. **d** Size of trans-Golgi network marked by TGN46 in HeLa cells treated with control or INPP5A siRNA (5AKD). Data are expressed in micrometer (length of long axis). Two-tailed paired *t*-test *p* = 0.0013, *t* = 6.474, df = 5. **e** Representative confocal images from five independent experiments of HeLa cells treated with control (SCR) or INPP5A siRNA (5AKD) and stained for PI(4)P (Red) and co-stained for the trans-Golgi marker TGN46 (green). Blue, DAPI-stained nuclei. Scale Bar, 10 μm. **f** Relative STX uptake into HeLa cells treated with either control siRNA (SCR, set to 1) or siRNA against OSBP (OSBP_KD). One sample *t*-test: *p* = 0.0035, *t* = 16.77, df = 2. **g**, **h** Relative STX uptake (**g**) STX surface level (**h**) into HeLa cells treated with either DMSO (set to 1) or the specific OSBP inhibitor OSW-1 (20 nM, 16 h). One sample *t*-test Uptake: *p* = 0.0007, *t* = 37.24, df = 2. Surface: *p* = 0.0002, *t* = 71.32, df = 2. **i** Relative STX uptake into wild-type (set to 1) or *VAP-A/B* double knockout (VAP_DKO) HeLa cells. One sample *t*-test: *p* = 0.006, *t* = 12.87, df = 2. Data represent mean ± SEM from three independent experiments in **a**, **b** **f**, **g**, **h**, **j** and from five independent experiements for **c** and six independent experiements in **d**. Numerical source data are reported in the Source Data file.

Based on these data we hypothesized that in the absence of INPP5A cholesterol fails to be properly transported from the ER to the Golgi complex leading to its partial depletion from the plasma membrane. Defective cholesterol export then results in the depletion of Gb3 from the plasma membrane and corresponding defects in CIE of Shiga toxin. Consistent with this hypothesis we observed that blockade of cholesterol biosynthesis in the presence of Mevastatin severely compromised surface binding (Fig. 5a, and Supplementary Fig. 3d) and internalization (Fig. 5b and Supplementary Fig. 3a) of Shiga toxin.

The transport of newly synthesized cholesterol from the ER, its site of synthesis, to the Golgi complex requires its oxysterol-binding protein (OSBP)-mediated transfer via VAP-containing membrane contact sites^11^. In this mechanism OSBP exchanges cholesterol present in ER membranes with phosphatidylinositol 4-phosphate [PI(4)P], a lipid highly enriched at the Golgi complex and the TGN^54^. As a result cholesterol is transferred from the ER to the TGN, while PI(4)P is transported retrogradely to the ER, where its phosphate is hydrolyzed by SAC1, thereby powering the OSBP cycle^11,54,55^. Loss of OSBP function has been shown to cause the accumulation of cholesterol at the ER and in lipid droplets and a concomitant rise in PI(4)P levels at the Golgi complex^11^. As loss of INPP5A also led to a partial redistribution of cholesterol to lysosomes and to ER-derived lipid droplets akin to inhibition of OSBP function (Fig. 4e, f), we probed whether this defect was accompanied by accumulation of PI(4)P in the Golgi compartment/TGN. Indeed, we observed profoundly elevated levels of Golgi/TGN-localized PI(4)P and a compaction of the TGN in INPP5A-depleted cells (Fig. 5c–e and Supplementary Fig. 4a). In contrast, the levels of PI(4,5)P_2_, a lipid predominantly localized to the plasma membrane and a potential substrate of INPP5A were slightly decreased in INPP5A knockdown cells (Supplementary Fig. 4b, c) (consistent with ref. ^42^). Cellular depletion of OSBP (Fig. 5f and Supplementary Fig. 3c, e), pharmacological inhibition of OSBP-mediated cholesterol/ PI(4)P exchange in the presence of the OSBP-specific small molecule inhibitor OSW-1^11^ (Fig. 5g, h and Supplementary Fig. 3b, d), or CRISPR/Cas9-mediated knockout of the OSBP binding partners *VAP-A and VAP-B*^56^(Fig. 5i and Supplementary Fig. 3e) phenocopied loss of INPP5A with respect to defective surface binding and CIE of Shiga toxin. Inhibition of OSBP-mediated cholesterol/ PI(4)P exchange by OSW-1 or KO of *VAP-A/B* also phenocopied INPP5A depletion with respect to reduced steady-state levels of SREBP-1 (Supplementary Fig. 4d–g).

Collectively, these results indicate that the reduced plasma membrane levels of Gb3 in INPP5A-depleted cells are a consequence of impaired OSBP-mediated cholesterol/PI(4)P exchange at ER/Golgi membrane contact sites. They further suggest that OSBP-mediated cholesterol export from the ER to the Golgi complex at MCS, a process regulated by INPP5A, is required for efficient Gb3 transport to or Gb3 stability at the plasma membrane.

### Receptor-mediated IP_3_-induced Ca^2+^ release represses CIE

**Fig. 6:**
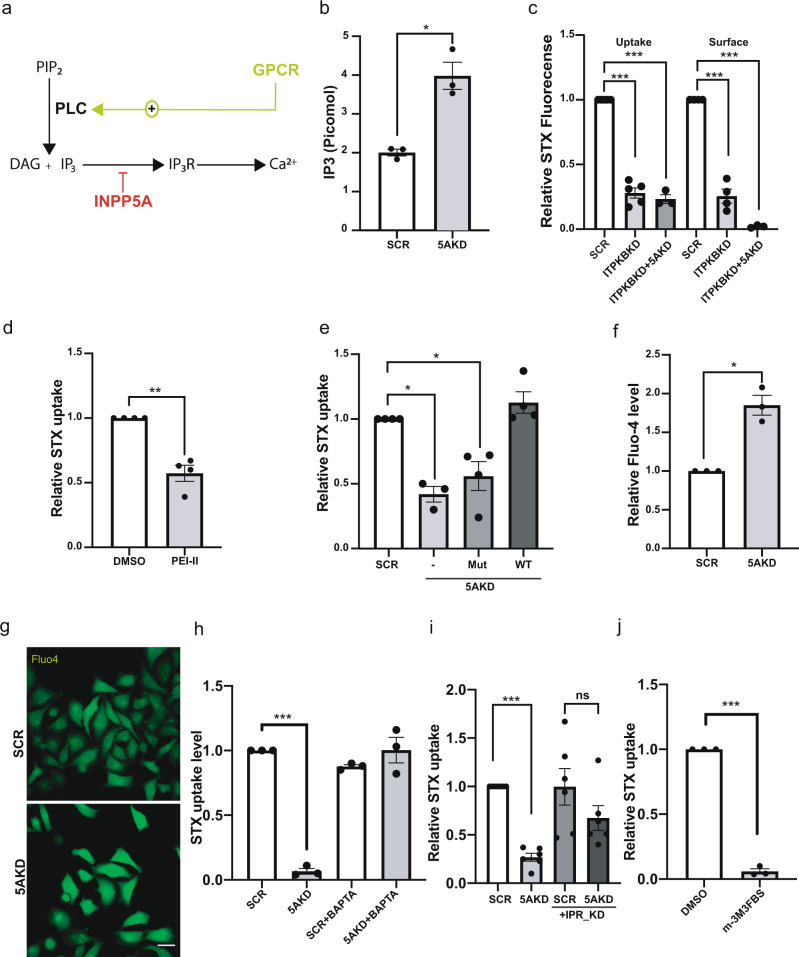
INPP5A controls surface lipid homeostasis by regulating IP_3_ levels. **a** Schematic of IP_3_ pathway. PLC, phospholipase C, DAG, diacylglycerol; IP3R, IP_3_ receptors. **b** [IP_3_] (picomole/500,000 cells) in HeLa cells treated with control or INPP5A siRNA (5AKD). Two-tailed paired *t*-test, *p* = 0.0265, *t* = 6.021, df = 2. Mean ± SEM, *n* = 3. **c** Surface binding and uptake of Shiga toxin (STX) in HeLa cells treated with the indicated siRNAs. Data for control set to 1. One sample *t*-test with Benjamini–Hochberg correction. Uptake ITPKBKD: *p* = 0.0002, *t* = 18.47, df = = 4. Uptake ITPKBKD + 5AKD: *p* = 0.0019, *t* = 23, df = 2. Surface ITPKBKD: *p* = 0.0002, *t* = 13.66, df =3. Surface ITPKBKD + 5AKD: *p* = 0.0008, *t* = 169.7, df = 2. Mean ± SEM, *n* = 3 (ITBKBKD + 5AKD), 4 (ITPKKD surface), or 5 (ITPKBD Uptake). **d** STX uptake in HeLa cells treated with PEI-II or DMSO. One sample *t*-test: *p* = 0.0064, *t* = 6.84, df = 3. Mean ± SEM, *n* = 4. **e** STX uptake in HeLa depleted of endogenous INPP5A expressing siRNA-resistant wild-type (WT) or phosphatase-dead (Mut) INPP5A. One sample *t*-test with Benjamini–Hochberg correction. -/5AKD: *p* = 0.0327, *t* = 9.492, df = 2. Mut/5AKD: *p* = 0.0.0435, *t* = 3.456, df = 2. Res/5AKD: *p* = 0.2225, *t* = 1.534, df = 3. Mean ± SEM, *n* = 3 (5AKD alone) or 4 (INPP5A transfections). **f**, **g** Relative Ca^2+^ levels determined by Fluo-4 in HeLa cells treated with control (SCR, set to 1) or INPP5A siRNA (5AKD). **g** Representative images, Scale bar, 25 μm. **f** Quantified data from *n* = 3. One sample *t*-test. 5AKD: *p* = 0.0217, *t* = 6.671, df = 2. **h** Rescue of defective STX uptake by BAPTA-AM. One sample *t*-test followed by Benjamini–Hochberg correction. 5AKD: *p* = 0.005, *t* = 42.70, df = 2. BAPTA/SCR: *p* = 0.0136, *t* = 8.488, df = 2. BAPTA/5AKD: *p* = 0.9762, *t* = 0.03365, df = 2. Mean ± SEM, *n* = 3. **i** Rescue of defective STX uptake by IP3R depletion. Relative STX uptake in HeLa cells treated with the indicated siRNAs. One sample *t*-test 5AKD: *p* < 0.0001, *t* = 17.94, df = 5. IPRsKD: *p* = 0.9866, *t* = 0.0176, df = 5. IPRsKS+5AKD = 0.0513, *t* = 2.55, df = 5 Mean ± SEM, *n* = 6. **j** PLC activation by m-3M3FBS inhibits STX uptake. One sample *t*-test m-3M3FBS: *p* = 0.0005, *t* = 46.51, df = 2. Mean ± SEM, *n* = 3. *n* denotes number of independent experiments, Numerical source data are reported in the Source Data file.

Next we aimed to unravel the pathway by which loss of INPP5A represses OSBP-mediated cholesterol export and, thereby, inhibit Gb3-dependent CIE of Shiga toxin. INPP5A has been been demonstrated to predominatly hydrolyze soluble IP_3_^34,42^ and, thus, may constitute a negative regulatory element in the signaling cascade that triggers IP_3_ receptor (IP_3_R)-mediated calcium release from the ER lumen via PLC-mediated cleavage of PI(4,5)P_2_ into diacylglycerol and IP_3_ downstream of receptor activation (Fig. 6a). We therefore hypothesized that elevated IP_3_ levels in absence of INPP5A might repress OSBP function, and, thereby, Shiga toxin CIE. We tested the putative role of IP_3_ in this pathway at multiple levels using CIE of Shiga toxin as a functional readout. First, we analyzed the cellular content of IP_3_. As expected, the levels of IP_3_ were increased about two-fold in INPP5A-depleted cells (Fig. 6b). Second, we reasoned that, if elevated IP_3_ levels were causative for defective Gb3-dependent Shiga toxin entry into INPP5A knockdown cells, other independent manipulations of IP_3_ turnover should phenocopy loss of INPP5A. IP_3_ can either be hydrolyzed to IP_2_ by inositol 5-phosphatases, most notably INPP5A, or be further phosphorylated by inositol triphosphate kinase B (ITPKB) to yield IP_4_^57,58^. We found that cellular depletion of ITPK reduced surface binding and internalization of Shiga toxin akin to INPP5A loss. Similar effects were seen upon co-depletion of both INPP5A and ITPKB (Fig. 6c and Supplementary Figs. 5a and 6g). To acutely elevate IP_3_ levels, we applied PEI-II, a blocker of prolyl oligopeptidase that indirectly stimulates IP_6_-to-IP_3_ conversion via multiple inositol phosphate phosphatase (MIPP)^59^. Application of PEI-II for 16 h also significantly reduced Shiga toxin CIE in genetically unperturbed wild-type HeLa cells (Fig. 6d and Supplementary Fig. 5b). Third, we probed whether the IP_3_-phosphatase activity of INPP5A is required for Shiga toxin endocytosis. Defective CIE of Shiga toxin was fully rescued by re-expression of active wild-type but not phosphatase-deficient INPP5A (Fig. 6e and Supplementary Fig. 5c, d). The impaired glycosphingolipid-dependent Shiga toxin CIE in the absence of INPP5A, thus, appears to be caused by elevated IP3 levels.

IP_3_ mainly acts by activating IP_3_ receptor (IP_3_R) channels in the ER to release calcium into the cytoplasm. Consistent with this, we found INPP5A-depleted cells to display increased cytosolic calcium levels monitored by Fluo4 (Fig. 6f, g). To probe whether elevated calcium levels were causal for the blockade of Shiga toxin CIE in absence of INPP5A, we sequestered intracellular calcium by application of the membrane-permeant chelator BAPTA/AM. This treatment fully rescued defective Shiga toxin-CIE in absence of INPP5A (Fig. 6h and Supplementary Fig. 5f). Sequestration of intracellular calcium via EGTA/AM also rescued impaired CIE of Shiga toxin (Supplementary Fig. 6d). We also perturbed IP_3_-induced calcium efflux from the ER by depleting HeLa cells of IP_3_Rs. Knockdown of *IP*_*3*_*R* expression by smart pool siRNAs (Supplementary Fig. 6e, f) also partially rescued impaired Shiga toxin endocytosis in INPP5A-depleted cells (Fig. 6i and Supplementary Fig. 5e). Finally, we perturbed the activation status of PLC, i.e., the enzyme that generates IP_3_ downstream of receptor signaling. Pharmacological activation of PLC by m-3M3FBS potently repressed CIE of Shiga toxin (Fig. 6j and Supplementary Fig. 6a). Conversely, impaired Shiga toxin uptake in INPP5A-depleted cells was partially reverted by PLC inhibition in the presence of D609 (Supplementary Fig. 6b, c). Hence, INPP5A controls glycosphingolipid-dependent CIE of Shiga toxin by hydrolyzing IP_3_, thereby counteracting IP_3_-induced calcium efflux via IP_3_Rs from ER stores downstream of signaling receptor activation.

### Ca^2+^ triggers OSBP dissociation from VAP-containing MCS

**Fig. 7:**
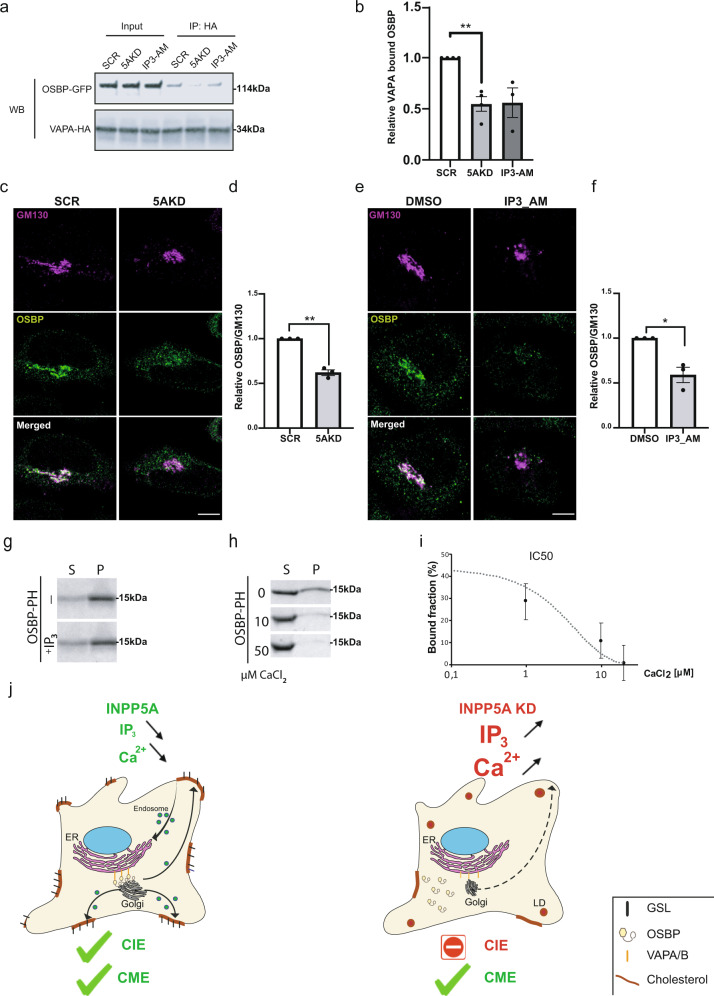
IP_3_-mediated Ca^2+^ release represses lipid exchange by inhibiting OSBP recruitment. (**a**) HEK-293T cells treated with either control siRNA (SCR) or siRNA against INPP5A (5AKD) or SCR-siRNA plus 50 μM of IP_3_/AM (2 h) and co-expressing VAP-A-HA and OSBP-GFP were lysed and subjected to immunoprecipitation with anti-HA antibodies coupled to magnetic beads Samples were analyzed by immunoblotting for HA or GFP. Input: 50 μg total cell lysate. **b** Quantification of OSBP-GFP co-precipitated with VAP-A-HA and normalized to total amount of VAP-A-HA bound to beads. Data for SCR-control conditions set to 1. Data are mean ± SEM from *n* = 4 independent experiments for 5AKD and *n* = 3 for IP_3_/AM. One sample *t*-test 5AKD: *p* = 0.0079, *t* = 6.353, df = 3. IP3-AM: *p* = 0.0927, *t* = 3,051, df = 2. **c**–**f** HeLa cells treated with control siRNA (SCR) or siRNA against INPP5A (5AKD) (**c**, **d**) or treated with DMSO or 50 μM of IP_3_/AM (**e**, **f**) were stained for GM130 (magenta) and OSBP (green). **c**, **e** Show representative images. Blue, DAPI-stained nuclei. Scale bar, 10 μm. **d**, **f** Relative OSBP level at the Golgi complex marked by GM130. Data are mean ± SEM from three independent experiments, control cells were set to 1, one sample *t*-test. 5AKD: *p* = 0.0071, *t* = 11.82, df = 2. IP3_AM *p* = 0.00415, *t* = 2.755, df = 2. **g**–**i** Liposome co-sedimentation assays to determine binding of purified recombinant OSBP-PH to PI(4)P-containing liposomes. S, supernatant; P, liposomal pellet. Samples were analyzed by SDS-PAGE and staining with Coomassie Blue. Representative data for effects of IP_3_ and Ca^2+^ are shown in **g** and **h**, respectively. **i** Bound liposome fraction and Ca^2+^ concentrations were plotted to determine the IC50 value, shown as the turning point at approximately 8 μM Ca^2+^. Data represent mean ± SEM from three independent experiments. **j** Hypothetical model for the role of INPP5A-mediated turnover of IP3 in the regulation of cholesterol export at ER-Golgi membrane contact sites and its consequences for cell physiology. Numerical source data and unprocessed blots are reported in the Source Data file.

These results suggest a model according to which the cytosolic concentrations of IP_3_ and/or calcium regulate OSBP-mediated cholesterol export at ER/Golgi MCS to control surface glycosphingolipid levels and, thereby, CIE of bacterial toxins. Given that OSBP-mediated lipid exchange requires the association of Golgi-localized OSBP with VAP proteins in the ER^13,54^, we hypothesized that INPP5A by controlling IP_3_ levels might impact on complex formation between VAP and OSBP proteins. Complex formation between OSBP-GFP and VAP-A-HA probed by co-immunoprecipitation experiments indeed was reduced by nearly 50% in INPP5A-knockdown cells, while OSBP expression was unaffected (Supplementary Fig. 7b, c). Importantly, a similar effect was observed in control HEK293-T cells, in which IP_3_ levels were acutely raised by application of cell membrane-permeant IP_3_/AM (Fig. 7a, b).

VAP is a transmembrane protein of the ER, whereas OSBP associates with the TGN via binding of its pleckstrin homology (PH) domain to PI(4)P^54,55^. Hence, we speculated that the accumulation of IP_3_ in the absence of INPP5A either directly or indirectly via IP_3_-induced calcium efflux from the ER might interfere with the recruitment and localization of OSBP to the TGN. Confocal imaging showed that while endogenous OSBP was concentrated in the TGN/Golgi area in close apposition to the Golgi marker GM130 in control cells, it adopted a largely dispersed localization in the cytoplasm of INPP5A-depleted cells (Fig. 7c, d). A similar, though even more striking loss of OSBP from TGN/Golgi membranes was observed in HeLa cells treated with cell membrane-permeant IP_3_/AM (Fig. 7e, f). Application of the calcium chelator EGTA/AM restored normal OSBP localization in INPP5A-depleted cells (Supplementary Fig. 7a). These data indicate that IP_3_ and/or calcium released from ER stores negatively regulate the association of OSBP with the TGN in living cells. Conceivably, the negative regulatory role of IP_3_ with respect to OSBP recruitment to the TGN could reflect competition of IP_3_ with PI(4)P via its charged phosphate-rich inositol headgroup, or be mediated indirectly via elevated calcium levels in the cytosol. For example, calcium could shield the headgroup of PI(4)P from recognition by the PH domain of OSBP^60^. To distinguish between these mechanisms, we assayed the recruitment of purified recombinant OSBP-PH domain to PI(4)P-containing liposomes with a Golgi-like lipid composition^61–63^ in vitro. Increasing concentrations of IP_3_ up to 700 μM, i.e., well above physiological IP_3_ levels, did not affect OSBP-PH binding to PI(4)P membranes (Fig. 7g), consistent with a role for IP_3_R-mediated calcium release from the ER lumen in the regulation of Shiga toxin CIE (compare Fig. 6). In contrast, OSBP-PH binding to PI(4)P-containing liposomes was potently inhibited by low micromolar calcium concentrations with an IC_50_ of about 8 μM (Fig. 7h, i), i.e., a value close to that reported for cytosolic calcium transients near IP_3_R sites^64–66^. These data suggest that calcium can directly interfere with the association of OSBP with PI(4)P-containing membranes (consistent with ref. ^67^) and, thereby, with its binding to VAP to promote lipid exchange. The association of the PH domain of the ceramide/PI(4)P lipid exchange protein CERT with PI(4)P-liposomes was much less affected by increasing calcium concentrations (Supplementary Fig. 7g, h), in agreement with the unaltered levels and distribution of ceramide (Fig. 2) in INPP5A knockdown cells.

Collectively, these results demonstrate that IP_3_-triggered calcium release blocks lipid exchange mediated by OSBP at ER-Golgi contact sites to regulate cholesterol and associated complex glycosphingolipid surface levels (Fig. 7j, model).

## Discussion

Our results reported here reveal a hitherto unknown function for IP_3_-triggered calcium release from the ER in the repression of cholesterol export at MCS between the ER and the TGN, a process controlled by the IP_3_-specific-phosphatase INPP5A (Figs. 4–6), an enzyme downregulated in cancer^34^, e.g., squamous cell carcinomas^33^, and spinocerebellar ataxia^37,68^. Defective cholesterol export at ER/ TGN MCS is further demonstrated to reduce the plasma membrane levels, but not or only moderately the total cellular content of the complex glycosphingolipid Gb3 (Figs. 1–3 and Supplementary Data File 1), likely as a consequence of its impaired assembly into transport-competent cholesterol-rich microdomains at the level of the Golgi complex^46^, resulting in a blockade of CIE of Shiga toxin (Figs. 1, 5,6). Finally, elevated cytosolic calcium levels induced by IP_3_-induced calcium release from ER stores in INPP5A-depleted cells are shown to inhibit membrane recruitment and localization of OSBP to the TGN via PI(4)P (Fig. 7).

This hypothetical pathway is supported by multiple lines of evidence: (i) Loss of INPP5A causes the selective accumulation of IP_3_ (Fig. 6), but not of its precursor and potential substrate PI(4,5)P_2_ from which it is derived (Supplementary Fig. 4b, c). The concomitant built-up of PI(4)P at the Golgi/TGN (Fig. 5) is explained by the observed mislocalization and loss-of-function of OSBP in INPP5A-depleted cells (Fig. 7). Consistent with this model, (ii) we observe that impaired CIE of Shiga toxin is rescued by WT but not phosphatase-inactive INPP5A that lacks the ability to hydrolyze IP_3_ or by co-depletion of IP_3_ receptors (Fig. 6), i.e., a condition that prevents IP_3_-mediated calcium efflux from the ER lumen. Furthermore, (iii) we show that exogenous application of membrane-permeant IP_3_ phenocopies loss of INPP5A with respect to impaired CIE of Shiga toxin and depletion of OSBP from the TGN and from VAP-containing complexes (Fig. 7), suggesting that the accumulation of IP_3_ is not only required but also sufficient to induce the cellular phenotypes characteristic of INPP5A loss. Fourth, (iv) we use quantitative lipidomic analyses by mass spectrometry (Figs. 2 and 3) to demonstrate that depletion of INPP5A does not alter total cellular lipid content including complex glycosphingolipids (i.e., receptor lipids for bacterial toxins such as Shiga toxin). Instead, Gb3 loss from the surface of INPP5A-depleted cells is shown to be an indirect consequence of defects in cholesterol export from the ER to the Golgi complex, which instead is rerouted to lysosomes and lipid droplets (Figs. 3 and 4), compartments that either emanate from the ER (i.e., lipid droplets) or are in contact with it via MCS (i.e., lysosomes). The key role of cholesterol in the maintenance of complex glycosphingolipids is further supported by the fact that acute blockade of cholesterol biosynthesis by Mevastatin, genetic or pharmacological inhibition of OSBP-mediated cholesterol transport out of the ER, or loss of ER/TGN MCS in *VAP-A/B* KO cells (Fig. 5) phenocopy INPP5A depletion with respect to loss of complex glycosphingolipids from the plasma membrane and defective CIE of Shiga toxin. Finally, (v) we demonstrate using purified components that low micromolar concentrations of calcium similar to those reported for cytosolic calcium transients near IP_3_R sites^64–66^, or reported to facilitate recruitment of E-Syts to ER-plasma membrane contact sites^5,7,38^ can directly interfere with the association of the PH domain of OSBP with PI(4)P-containing membranes (Fig. 7). These data are consistent with a direct effect of calcium on OSBP localization and, thereby, VAP association, although additional indirect roles of calcium in the regulation of OSBP localization and MCS formation, e.g., via calcium-regulated kinases or phosphatases, cannot be ruled out. In contrast to the effects of calcium or INPP5A loss on membrane association of OSBP, we observed only minor effects of INPP5A knockdown on the localization of the PI(4)P-binding glucosylceramide transfer protein FAPP2^69^ at the TGN (Supplementary Fig. 7d–f).

At the moment, we can only speculate about the exact mechanism by which impaired cholesterol export from the ER to the TGN under conditions of INPP5A or VAP loss causes the depletion of Gb3 from the cell surface. We note that previous studies have established a tight physical and functional connection between cholesterol and complex glycosphingolipids such as Gb3, e.g., as constituents of microdomains in the Golgi complex^46^, as GPI-linked signalling complexes, or as key regulators of endocytic tubule formation that mediate cell entry of bacterial toxins including Shiga toxin^17,18,23–25,28^. We, thus, hypothesize that defective cholesterol export to the TGN affects Gb3 at the level of the Golgi complex, where it is synthesized. For example, it is possible that under conditions of impaired cholesterol export from the ER in INPP5A-depleted, OSBP-depleted, or VAP-depleted cells Gb3 following its biosynthesis fails to assemble into stable microdomains in the late Golgi^46^ and, therefore, fails to be transported to the plasma membrane. Future studies will be needed to explore this mechanism in more detail.

Our findings also expand previous studies on the regulation of MCS by calcium signaling^5,7,8^. Distinct from the established function of cytoplasmic calcium signals in triggering STIM1-ORAI channel formation^9,29^ and the recruitment of ER-localized E-Syts to ER-plasma membrane contact sites, we observe an inhibitory role of calcium with respect to OSBP/ VAP-mediated lipid exchange at ER-TGN contacts. E-Syts are ER-localized C2 domain proteins that bind to the plasma membrane in *trans* via recognition of PI(4,5)P_2_ by their so-called C2 domains. In the case of E-Syt1 micromolar concentrations of calcium, e.g., induced by receptor signaling via PLC-triggered IP_3_-induced calcium release from ER stores^38^, promote its association with the plasma membrane to transfer glycerolipids such as diacylglycerol^4,5^, between the plasma membrane and the ER. We now show that a similar pathway via IP_3_-induced calcium release from the ER (Fig. 6) represses the exchange of cholesterol and PI(4)P at ER/ TGN contact sites by calcium-mediated inhibition of OSBP recruitment to PI(4)P-containing TGN membranes. It therefore appears that signaling via PLC-coupled receptors differentially affects lipid exchange via E-Syts at ER/plasma membrane vs. OSBP at ER/TGN contact sites. It is conceivable that the physiological effects of IP_3_-induced calcium release from the ER^8,29^ may also affect non-vesicular lipid transport at other intracellular MCS, that depend on phosphosphoinositide-based membrane tethers, for example ER-endosome^56,70^ or ER-lysosome contacts^3,4,6^. Given that several types of MCS play key roles in calcium channeling and homeostasis^8^ it is tempting to speculate that MCS formation and function may be part of a feedback-regulated molecular network, that serves to integrate lipid flux with signalling-induced fluctuations in calcium levels^8,29^. Whether and how this network impinges on the IP_3_-phosphatase INPP5A itself, is unclear. Previous work had suggested that INPP5A may be regulated by phosphorylation-dependent association with inhibitory 14-3-3 proteins^71^. Our attempts to determine the precise localization and mode of regulation of endogenous INPP5A have been unsuccessful so far, largely owed to its low levels of expression.

We further show that loss of INPP5A and the resulting depletion of plasma membrane cholesterol and Gb3 from the cell surface potently blocks CIE of Shiga toxin. Glycosphingolipids, such as Gb3, also play an important roles in other forms of CIE such as the internalization of CD44 and integrins via the CLIC/GEEC pathway^22^, which may conceivably be regulated by IP_3_ signaling and turnover mediated by INPP5A. CIE pathways similar to the Shiga toxin route of entry control the surface levels and activity of PLC-linked cell signalling receptors^26–28,72^. In this context, it is interesting to note that reduced expression of INPP5A and changes in the synthesis, and/or surface expression of glycosphingolipids and cholesterol have been associated with cancer and metastasis^16,20^, processes regulated by the exo-endocytosis of cell signaling and adhesion receptors such as integrins, and with regulated cell death by apoptosis^73^. Reduced expression of INPP5A has also been found to be involved in spinocerebellar ataxia^37,68^, a group of neurodegenerative diseases that includes forms intimately linked to defective Golgi-to-plasma membrane trafficking^74,75^. Exploring these mechanisms in detail remains a fruitful area for future studies.

## Methods

### siRNAs

siRNA oligonucleotides used in this study (SMART pools consisting of 4 siRNAs) are listed in Supplementary Table 1.

### Antibodies

Antibodies used in this study are listed in Supplementary Table 1. Antibodies used for immunoblotting and immunocytochemistry. Secondary antibodies for immunoblotting were either Peroxidase-conjugated for horse radish peroxidase (HRP)-detection or conjugated to a fluorescent dye for fluorescence detection. Secondary antibodies for immunocytochemistry were Alexa Fluor (AF)-conjugated. ICC immunocytochemistry, IB immunoblotting, rb rabbit, ms mouse, r rat, gt goat, dk donkey, AF Alexa Fluor, α anti, H & L heavy and light chain.

### Plasmids

pcDNA3.1(+), EcoRV/XbaI-INPP5Awt, pcDNA3.1(+),EcoRV/XbaI-INPP5Apase dead, were designed with GeneArt services from ThermoFisher. D4Hmcherry-pGEX-6P1 is a gift from Pr. Gregory D Fairn. pEGFP-N1-OSBP is a gift from Pr. Pietro De Camilli and pGW-VAP-HA was a gift from Pr. Caspar C. Hoogenraad. Green fluorescent protein (GFP)–FAPP2 wild-type was a gift from Pr. Maria Antonietta De Matteis.

### Quantitative real-time RT-PCR (qPCR)

Cultures were harvested and total RNA extracted using a RNeasy Mini kit (QIAGEN). The concentration of RNA was measured with a spectrophotometer (Nano Drop) and 500 ng of RNA per sample were used in each reaction according to instruction in SuperScript™ IV First-Strand Synthesis System (ThermoFisher) to create cDNA library. Hundred nanogram of cDNA were used in each reaction according to SsoAdvanced Universal SYBR® Green Supermix (QIAGEN). Samples were subsequently loaded on to StepOnePlus™ Real-Time PCR System (ThermoFisher). Ct values were obtained and converted to relative mRNA expression levels. GAPDH was used as a reference/normalization control. Primer sequences for qPCR are are listed in Supplementary Table 2.

### Chemicals and inhibitors

All chemicals and inhibitors were dissolved according to the manufacturer’s instructions to the indicated concentrations in the appropriate solvent. Working concentrations are indicated for each experiment. OSW-1 targeting OSBP was prepared in a stock solution of 2 µM and purchased from BOC Sciences (B0005-092456). Bt3-Ins(145)P3/AM was prepared at 100 mM stock solution and purchased from Sichem (3-1-145). PEI-II was prepared at 24 mM stock solution and purchased from Sigma-Aldrich (537011). Mevastatin-Sodium Salt was prepared at 30 mg/ml stock solution and purchased from Sigma-Aldrich (474705), GM1 was purchased from Sigma-Aldrich (G7641). D609 purchased from TOCRIS (1437) was prepared as a 10 mM stock solution. m-3M3FBS was purchased from Sigma-Aldrich (525185) and prepared as a 10 mM stock solution in DMSO. EGTA-AM was prepared at 50 mM stock solution and purchased from Merck (324628). BAPTA, AM, cell permeant chelator was prepared at 1 mM stock solution and purchased from ThermoFischer (B6769).

### Probes

We purchased from Sigma-Aldrich; Shiga Toxin Subunit B (SML0562) and prepared a stock solution of 100 µg/ml. Filipin III (F4767) and prepared it at 25 mg/ml. We purchased from Thermo-Fisher; BODIPY (D3922) and prepared it at 2 mM. EGF-Alexa Fluor 647 (E35351) and prepared it at 40 μg/ml. Tf-Alexa Fluor 647 (T23366) and prepared it at 5 mg/ml. WGA Alexa Fluor 647 (W32466) and prepared it at 1 mg/ml. Fluo-4 (F14201) and prepared it at 1 mM. LysoTracker™ Red DND-99 (L7528) and LysoTracker™ Green DND-26 (L7526) both were purchased from ThermoFisher and made at 1 mM stock solution each.

### Cells

HeLa, HeLaM, and HEK293T and COS7 cells were obtained from the American Type Culture Collection. Control. *VAP-A/B* double knock-out^56^ and parental HeLa cells were a gift from Prof Pietro De Camilli’s laboratory (Yale University School of Medicine, New Haven, CT, USA). Cells were cultured in DMEM with 4.5 g/l glucose (Lonza) containing 10% heat-inactivated FBS (Gibco) and 100 U/ml penicillin and 100 μg/ml streptomycin (Gibco) during experimental procedures. Human *INPP5A* 4 bp deletion KO cell line and a C631 human HAP1 parental control cell line was purchased from Horizon Discovery (HZGHC006619c008). Cells were routinely tested for Mycoplasma contamination and all tests were negative.

### siRNA and cDNA transfections

100,000 (Hela, HeLaM, COS-7) cells or 300,000 HEK293T cells, with low passage numbers, are reverse-transfected using jetPRIME transfection reagent according to the manufacturer protocol using 100 nM of each siRNA. If DNA cotransfection is needed, cells are first reverse-transfected with siRNA, and 24 h later, 2 µg of DNA is transfected using the jetPRIME protocol.

### Inositol-1,4,5-trisphosphate measurements

IP_3_ was determined using the inositol-1,4,5-trisphosphate [^3^H] radioreceptor assay kit from Perkin Elmer (NEK064). Briefly, 500,000 HeLa cells were treated with control siRNA or siRNA against INPP5A for 48 h. Cells were washed with PBS, trypsinized, and collected into 500 ml fresh medium before being placed on ice. Immediately thereafter 100 ml of ice-cold 100% trichloroacetic acid (TCA) solution was added. Samples were vortexed thoroughly and incubated in ice for 15 min. Samples were centrifuged for 10 min at 4 °C at 1000×*g* and the supernatant was collected. TCA was removed from samples using TCTFE-trioctylamine (2 ml for each 1 ml of TCA) and the upper phase was collected. A working receptor/[^3^H]IP_3_ tracer solution was prepared, 100 μl of each sample or diluted standard was added to 400 μl of the working receptor/tracer solution. After 1 h of incubation in ice samples were centrifugated 15 min at 1500×*g*. Fifty microliter of 0.15 M sodium hydroxide was added to each tube and incubated for 10 min at room temperature, mixed with 5 ml of Formula-989 scintillation cocktail before placing the vials into a scintillation counter. The amount of [^3^H]IP_3_ was determined by incubating the supernatant with a membrane preparation from calf cerebellum containing the IP_3_ receptor. The addition of unlabeled IP_3_ to the incubation mixture as either a standard or unknown sample, competes with [^3^H]IP_3_ for binding to the receptor and lowers the amount of radioactivity in the membrane pellet.

### PH domain expression and purification

The PH-domain of OSBP (87–190) and of CERT (24–117) were cloned into a pETet28a(+) Vector and expressed in Escherichia coli BL21 DE3 cells. Bacteria were cultured in 2×YT medium at 37 °C to an OD600 of 0.6 followed by a temperature shift to 18 °C. Protein was expressed for 18 h by adding 200 μM isopropyl β-d-1-thiogalactopyranoside. Cells were harvested by centrifugation, resuspended in lysis buffer containing 50 mM HEPES pH 7.5, 500 mM NaCl, 2 mM DTT, 1 μM DNase (Roche), and 100 μM Pefabloc (Roth), and lysed by sonication. Lysates were cleared by centrifugation at 40,000×*g* for 20 min at 4 °C. The supernatant was applied to a 1 ml Ni-NTA column pre-equilibrated with 50 mM HEPES/NaOH pH 7.5, 500 mM NaCl, 20 mM imidazole, 2 mM DTT. The column was extensively washed with this buffer. His6-tagged protein was eluted with 50 mM HEPES/NaOH pH 7.5, 500 mM NaCl, 300 mM imidazole, 2 mM DTT, and dialyzed against 50 mM HEPES/NaOH pH 7.5, 150 mM NaCl, 5 mM DTT overnight at 4 °C. The protein was concentrated and flash frozen in liquid nitrogen.

### Liposome co-sedimentation assay

Liposomes were produced by mixing PC:PE:SM:Chol:PI(4)P (Sigma and Avanti Lipids) in a molar ratio of 50:20:6:20:1.5 followed by drying under Argon stream and solubilization in 20 mM HEPES/NaOH pH 7.5, 150 mM NaCl. Liposomes at a final concentration of 1 mg/ml were incubated with 20 μM protein for 10 min at room temperature in a 40 μl reaction volume, followed by a 200,000×*g* spin for 10 min at 20 °C (see also http://www.endocytosis.org). The liposome binding competition assay was performed at final concentration of 10, 20, and 50 µM CaCl_2_ or 700 µM IP3 (1,4,5; Sigma) dissolved in Liposome solubilization buffer. The intensity of the SDS bends were quantitatively analyzed using the Image Lab Software suite (BioRad).

### Shiga toxin uptake assays

Upon siRNA revers transfection, cells were plated on glass coverslips to reach 70–80% confluency on the day of the experiment Alexa Fluor647-labeled Shiga toxin (final concentration 5 µg/ml) was diluted in the serum free DMEM and centrifuged for 5 min at 18,200×*g* to clarify the solution. Cells were incubated with Shiga toxin for 45 min at 37 °C (coverslips upside down), washed three times with PBS and fixed using 4% PFA/4% sucrose for 30 min at RT. Images were acquired with EPI microscope and Fluorescence intensities per cell were quantified using ImageJ.

### EGF/transferrin/wheat germ agglutinin (WGA) surface labelling and uptake assays

Cells were plated on glass coverslips to reach 70–80% confluency on the day of the experiment. Cells were starved in serum free DMEM for 1 h and 3 h, respectively, but for WGA surface labelling cells were not starved. For surface labelling cells (coverslips upside down) were incubated with Tf-Alexa Fluor 647 (25 µg/ml in serum-free DMEM), EGF-Alexa Fluor 647 (500 ng/ml in serum-free DMEM) and WGA Alexa Fluor 647 (2.5 µg/ml in HBSS) for 45 min at 4 °C (incubation chambers on ice in the cold room), washed three times with ice cold PBS. For Tf and EGF uptake, cells were incubated with Tf-Alexa Fluor 647 (25 µg/ml) and EGF-Alexa Fluor 647 (500 ng/ml) for 10 min (Tf) or 30 min (EGF) at 37 °C. Next, cells were washed with ice cold PBS and non-endocytosed ligand, still bound to the surface, was removed by acid wash (0.2 M NaCl, 0.1 M NaOAc pH 5.3, 1 min on ice). Cells were washed twice with ice cold PBS. All samples were fixed using ice-cold 4% PFA/4% sucrose for 45 min at RT. Images were acquired with EPI microscope and Fluorescence intensities per cell were quantified using ImageJ.

### Isolation of plasma membrane-enriched fractions from HeLa cells

To isolate the plasma membrane, the MinuteTM Plasma Membrane Protein Isolation and Cell Fractionation Kit (SM-005) from (Invent Biotechnologies) was used. For each condition, three biological samples were prepared: one technical replicate was prepared for subsequent western blot analysis. To separate the membrane HeLa cells, 5 × 10^6^ cells were used per sample. Cells were harvested by spinning them down at 300×*g* for 5 min. Then, the cells were resuspended in ice cold PBS and centrifuged at 1700×*g* and 4 °C for 1 min to remove residual culture medium. After PBS was removed completely, the cells were sensitized for lysis in 500 µl Buffer A supplemented with 1× Roche complete protease inhibitor cocktail (PIC). Suspension was mixed on the Vortex shaker for 20 s and incubated for 10 min on ice. Then, the suspension was transferred to the filter cartridges and centrifuged for 30 s at 16,000×*g* and 4 °C. To increase the yield, the resulting pellet was resuspended in the flow through and passed through the filter a second time with the same conditions. For separation of nuclei from membrane and cytosolic fractions, the ruptured cells were centrifuged at 700×*g* and 4 °C for 1 min. The supernatant was transferred to a new reaction tube and centrifuged for 30 min at 16,000×*g*, 4 °C. the resulted supernatant contained the cytosolic proteins, while the total membrane proteins were collected in the pellet. The total membrane protein pellet was resuspended in 200 µl Buffer B by pipetting and shaking with the Vortex mixer. To separate organelle membrane proteins from plasma membrane proteins, samples were centrifuged at 7800×*g* and 4 °C for 20 min. The pellet containing organelle membrane proteins was stored undissolved at − 20 °C until further use. Supernatant was transferred to a new 2.0 ml reaction tube and combined with 1.6 ml cold PBS by inversion. Centrifugation was conducted at 16,000×*g* for 30 min and the pellet containing the plasma membrane proteins was stored at −80 °C until lipidomics analysis.

### Lipid analysis of cells and plasma membrane-enriched fractions

Cells were subjected to lipid extractions using an acidic liquid-liquid extraction method^76^, except for plasmalogens, which were extracted under neutral conditions.

To ensure that similar amounts of lipids were subjected to extractions, a test extraction was performed to determine the concentration of PC as a bulk membrane lipid and to adapt extractions volumes to similar total lipid amounts. Typically, a total lipid amount of approximately 2600 pmol (cells) or 2900 pmol (subcellular fractions) was subjected to extractions. Quantification was achieved by adding 1–3 internal lipid standards for each lipid class, with the standards resembling the structure of the endogenous lipid species. Of note, sample volumes were adjusted to ensure that all lipid standard to lipid species ratios were in a linear range of quantification. Typically, the range of standard to species ratios were within a range of >0.1 to <10. Following this approach, a relative quantification of lipid species was performed. Lipid standards were added prior to extractions, using a master mix consisting of 50 pmol phosphatidylcholine (PC, 13:0/13:0, 14:0/14:0, 20:0/20:0; 21:0/21:0, Avanti Polar Lipids), 50 pmol sphingomyelin (SM, d18:1 with N-acylated 13:0, 17:0, 25:0, semi-synthesized^77^, 100 pmol deuterated cholesterol (D_6_-cholesterol or D_7_-cholesterol, Cambridge Isotope Laboratory), 30 pmol phosphatidylinositol (PI, 17:0/20:4, Avanti Polar Lipids), 25 pmol phosphatidylethanolamine (PE) and 25 pmol phosphatidylserine (PS) (both 14:1/14:1, 20:1/20:1, 22:1/22:1, semi-synthesized^77^), 25 pmol diacylglycerol (DAG, 17:0/17:0, Larodan), 25 pmol cholesteryl ester (CE, 9:0, 19:0, 24:1, Sigma), and 24 pmol triacylglycerol (TAG, LM-6000/D5-17:0,17:1,17:1, Avanti Polar Lipids), 5 pmol ceramide (Cer, d18:1 with N-acylated 14:0, 17:0, 25:0, semi-synthesized^77^ or Cer d18:1/18:0-D3, Matreya) and 5 pmol glucosylceramide (HexCer) (d18:1 with N-acylated 14:0, 19:0, 27:0, semi-synthesized or GlcCer d18:1/17:0, Avanti Polar Lipids), 5 pmol lactosylceramide (Hex2Cer, d18:1 with N-acylated C17 fatty acid), 10 pmol phosphatidic acid (PA, 17:0/20:4, Avanti Polar Lipids), 10 pmol phosphatidylglycerol (PG, 14:1/14:1, 20:1/20:1, 22:1/22:1, semi-synthesized^77^) and 5 pmol lysophosphatidylcholine (LPC, 17:1, Avanti Polar Lipids). The phosphatidylethanolamine plasmalogen (pl-PE-) standard mix consisted of 16.5 pmol PE P-Mix 1 (16:0p/15:0, 16:0p/19:0, 16:0p/ 25:0), 23.25 pmol PE P-Mix 2 (18:0p/15:0, 18:0p/19:0, 18:0p/25:0), 32.25 pmol PE P-Mix 3 (18:1p/15:0, 18:1p/19:0, 18:1p/25:0). Semi-synthesis of PE P- was performed as described in the ref. ^78^. The final CHCl_3_ phase was evaporated under a gentle stream of nitrogen at 37 °C. Samples were either directly subjected to mass spectrometric analysis, or were stored at −20 °C prior to analysis, which was typically done within 1–2 days after extraction. Lipid extracts were resuspended in 10 mM ammonium acetate in 60 µl methanol. Two microliter aliquots of the resuspended lipid extracts were diluted 1:10 in 10 mM ammonium acetate in methanol in 96-well plates (Eppendorf twin tec 96) prior to measurement. For cholesterol determinations, the remaining lipid extract was again evaporated and subjected to acetylation as described in the ref. ^79^. Samples were analyzed on an QTRAP6500+ (Sciex) mass spectrometer, except for cholesterol determinations, which were done on a QTRAP5500 (Sciex), with chip-based (HD-D ESI Chip, Advion Biosciences) electrospray infusion and ionization via a Triversa Nanomate (Advion Biosciences) as described. MS settings and scan procedures are described in Supplementary Data File 1. Data evaluation was done using LipidView (Sciex) and an in-house-developed software (ShinyLipids). The amount for endogenous molecular lipid species was calculated based on the intensities of the internal standards. For the quantification of GM1, GM2, GM3, and GB3, extractions were performed by a 2-step procedure as described in the ref. ^80^. Briefly, sample amounts of approx. Fifteen to twenty nanomolar of total lipid were subjected to the glycosphingolipid analysis. The sample volumes were adjusted to 200 µl using 155 mM ammonium bicarbonate in methanol. The first neutral extraction was performed using chloroform:methanol (17:1, vol:vol), followed by a chloroform:methanol (2:1, vol:vol) extraction. The following internal standards (Matreya LLC) were used: 25 pmol GB3 (d18:1/18:0-D_3_), 25 pmol GM3 (d18:1/18:0-D_3_), 25 pmol GM2 (d18:1/18:0-D_3_), and 25 pmol GM1 (d18:1/18:0-D_3_) semi-synthesized in-house, following a protocol described in the ref. ^81^. For cells, residual amounts of GM1 were recovered from the aqueous phase of the 2-step extraction by a C8 functionalized solid-phase extraction^82^. C8-cartdriges were equilibrated in methanol and conditioned with 60% methanol in water. Samples were loaded in 60% methanol in water and the first flowthrough was reapplied. The column was washed with 60% methanol in water and eluted in methanol. Following evaporation of the organic solvent, lipid extracts were resuspended in 100 µl mobile phase containing 60% of mobile phase A (acetonitrile:water, 60:40 (vol:vol) in 10 mM ammonium formate and 0.1% formic acid) and 40% mobile phase B (isopropanol:acetonitrile, 90:10 (vol:vol) in 10 mM ammonium formate and 0.1% formic acid), and transferred to silanized glass inlets in 2 ml glass vials with PTFE-coated caps. Glycosphingolipids were subjected to UHPLC-MS analysis at a flow rate of 0.1 ml/min, using a CSH C18 column (1 × 150 mm, 1.7 µm particles, Waters) coupled to a QExactive Q/orbitrap tandem-MS (Thermo Scientific) equipped with an ESI source. Measurement in positive and negative ion mode was performed including switching between Full-MS and All ions fragmentation (AIF). For one injection 12.5 µl of each sample was subjected to UPLC separation (Dionex), using a step gradient of 60–50% A (0.0–3.0 min), 50–46% A (3.0–9.0 min), 46–30% A (9.0–9.1 min), 30–10% A (9.1–17.0 min), 10% A (17.0–22.0 min), 10–60% A (22.0–22.5 min), and 60% A (22.5–30.0 min). The column temperature was set to 55 °C and the flow rate to 0.1 ml/min. Full MS scans were acquired for 25 min in positive and negative ion mode (*m*/*z* 500–1800) were with automatic gain control (AGC) target of 3 × 10^6^ ions, maximal injection time of 200 ms and resolution set to 140,000. AIF in positive and negative ion mode was performed with AGC target of 1 × 10^6^ ions, scanning a mass range of 120–600 *m*/*z* with normalized collision energy set to 30 eV.

Data evaluation of Full-MS scans (profile spectra) was performed using MassMap® (MassMap, Germany). Data files were converted to mzXML-files using the software MSconvert (Proteowizard). mzXML-Files were then converted to mmp-Files using the MassMap software. Individual scans in positive, negative, or Full-MS and AIF mode were filtered for further processing. Instrument noise in acquisitions was subtracted in the mmp-files. A target mass list was generated, consisting of the name of the target molecules, the monoisotopic mass and the average mass of the target compound. Applying this list, the software selects for chromatographic peaks, matching spectra with a predefined mass deviation. Automatic hits were validated in a user assessment. Amount of target compounds were quantified by R-packages and MS excel.

### Light microscopy of cultured cells

Treated cells were washed once with PBS, fixed with 4% PFA/4% sucrose for 20 min at RT. Washed three times with PBS-10 mM MgCl_2_ and blocked/permeabilized with GSDB [10% Goat serum, 100 mM NaCl, 0.3% Triton X-100, in PBS] for 30 min at RT. Primaries antibodies were diluted in GSDB and applied for 2 h at RT. Next cells were washed three times with PBS-10 mM MgCl_2_ and incubated with appropriate Alexa Conjugated secondary antibodies diluted in GSDB. Cells were washed three times with PBS-10 mM MgCl_2_, dipped in the water and mounted with immunomount supplemented with DAPI. Images were acquired with a minimum resolution (512px × 512px) for confocal imaging using Zeiss LSM 710 or LSM780 microscopes. Images analysis and quantification were performed using FIJI.

### Measurements of intracellular calcium levels

Calcium levels at the steady state were measured in HeLaM cells treated with control or INPP5A siRNA. Cells were incubated with 2 mM Fluo-4/0.02% pluronic for 10 min in serum free DMEM. Then cells were washed three times with PBS supplemented with calcium and Mg^2+^. Cells were imaged life in PBS and images were acquired with LSM.

### Phosphoinositide staining

Cells were washed with PBS-10 mM MgCl_2_, fixed with 2% PFA/2% sucrose/PBS for 20 min and permeabilized with 0.5% Saponin (in PBS/1% BSA) for 30 min at RT. Then cells were incubated with anti-PI(4)P or PI(4,5)P_2_ antibodies diluted in PBS/1% BSA for 2 h at RT. After three washes with PBS-10 mM MgCl_2_, cells were incubated with goat anti mouse igM AF568 diluted in PBS/1% BSA. Cells were washed three times with PBS-10 mM MgCl_2_ and mounted with immomount supplemented with DAPI.

### Lipid droplet staining

cells were revers transfected with negative control siRNA or siRNA against INPP5A, then seeded over a Matrigel-coated cover glass for 48 h and incubated in 5% CO_2_ at 37 °C. Then cells were washed twice with PBS and incubated with for 15 min with 2 μM BODYPI 493/503 (4,4-Difluoro-1,3,5,7,8-Pentamethyl-4-Bora-3a,4a-Diaza-s-Indacene) before they are fixed with 4% PFA/4% sucrose for 30 min at room temperature (RT). Cells were washed three times with PBS-10 mM MgCl_2_ and mounted with immomount supplemented with DAPI.

### Filipin staining

HeLa cells were transfected with negative control siRNA or siRNA against INPP5A, then seeded onto Matrigel-coated cover glass for 48 h and incubated in 5% CO_2_ at 37 °C. Cells were washed twice with PBS and fixed with 4% PFA/ 4% sucrose for 60 min at RT. Cells were washed three times with PBS and incubated with 1.5 mg/ml glycine in PBS for 10 min, then incubated in a dark room with the Filipin working solution for 2 h. Samples were rinsed three times with PBS and imaged live by epifluorescence microscopy using a UV filter set (340–380 excitation, 40 nm dichroic, 430-nm long pass filter).

### Immunoprecipitation

HEK293T cells were co-transfected with OSBP-GFP and VAPA-HA in combination with negative control siRNA or with siRNA against INPP5A or negative control siRNA. Where indicated, HEK293T cells were treated for 2 h with 50 μM IP3_AM. Cells were washed three times in PBS and lysed in immunoprecipitation (IP) buffer (20 mM HEPES, pH 7.4, 130 mM NaCl, 0.3% protease inhibitor cocktail (Sigma-Aldrich), 10 mM NaF, phosphatase inhibitors (cocktails 2 and 3, Sigma-Aldrich) and 1% NP-40). Samples were centrifuged at 10,000×*g* for 10 min at 4 °C, and the supernatant was then incubated with HA-Trap magnetic microparticles (ChromoTek) for 1 h at 4 °C. Beads were washed three times with IP buffer, and bound proteins were eluted in SDS–PAGE sample buffer, resolved by SDS–PAGE and analysed via immunoblot.

### Cell lysates and immunoblotting

Cells were washed three times in ice-cold PBS and collected in PBS with 1% Triton X-100, 0.3% protease inhibitor cocktail (Sigma-Aldrich) and phosphatase inhibitors (cocktails 2 and 3, Sigma-Aldrich). Protein levels were quantified using Bradford reagent (Sigma-Aldrich). Equal concentration lysates in Laemmli sample buffer were boiled for 5 min; between 10 and 50 μg protein was resolved by SDS-PAGE and analysed via immunoblot using LI-COR 800CW and 680RD infrared secondary antibodies as indicated. Immunoblots were imaged and quantified using Odyssey software.

### Statistics and reproducibility

Values are depicted as mean ± SEM or mean ± SD as indicated in the figure legends. One-sample two-sided *t*-tests were used for comparisons with control group values that had been set to 1 for normalization purposes and that therefore did not fulfill the requirement of two-sample *t*-tests or one-way ANOVA concerning the homogeneity of variances. The Benjamini–Hochberg procedure was used to correct for multiple testing based on the acceptance of a false discovery rate of 5% (see figure legends). GraphPad Prism version 8 software was used for statistical analysis. The level of significance is indicated in the figures by asterisks (*p ≤ 0.05; *p ≤ 0.01; ***p ≤ 0.001; ****p ≤ 0.0001) and provided in the figure legends as exact *p*-value as obtained by the indicated statistic test. No statistical method was used to pre-determine sample size as sample sizes were not chosen based on pre-specified effect size. Instead, multiple independent experiments were carried out using several sample replicates as detailed in the figure legends.

### Reporting summary

Further information on research design is available in the Nature Research Reporting Summary linked to this article.

## Supplementary information

Supplementary Information

Description of Additional Supplementary Files

Supplementary Data 1

Reporting Summary

## Data Availability

The data that support these findings are available from the authors on request. All lipidomics data are reported Supplementary Data File 1 and have been deposited to the Metabolights database, where they are available under accession number MTBLS2444. Numerical source data for Figs. 1–7 and uncropped versions of blots and gels are provided in the Source Data File. Requests for materials should be addressed to V.H. (haucke@fmp-berlin.de). Source data are provided with this paper.

## Source data

Source Data
