# Supplementary material for: Inositol triphosphate-triggered calcium release blocks lipid exchange at endoplasmic reticulum-Golgi contact sites
Source: Nat Commun. 2021 May 11;12:2673. doi: 10.1038/s41467-021-22882-x (PMC8113574; doi:10.1038/s41467-021-22882-x)
Supplement: Supplementary file 1 — Supplementary Information [file 41467_2021_22882_MOESM1_ESM.pdf]

**Supplementary Information for**

# Inositol triphosphate-triggered calcium release blocks lipid exchange at endoplasmic reticulum-Golgi contact sites

Mouhannad Malek, Anna M. Wawrzyniak, Peter Koch, Christian Lüchtenborg, Manuel Hessenberger, Timo Sachsenheimer, Wonyul Jang, Britta Brügger, Volker Haucke

## Inventory of supplementary items

**Supplementary figures**

Supplementary figure 1 | INPP5A loss impairs clathrin-independent endocytosis of bacterial Shiga toxin but not CME.
Supplementary figure 2 | Cholesterol rerouting to lysosomes and lipid droplets in absence of INPP5A.
Supplementary figure 3 | | Cholesterol depletion or loss of VAP or OSBP proteins impair CIE of Shiga toxin.
Supplementary figure 4 | Levels of PI(4,5)$P_2$ and Golgi PI(4)P in INPP5A-depleted cells and effects of VAP or OSBP loss-of-function on SREBP-1 protein expression.
Supplementary figure 5 | INPP5A controls Shiga toxin endocytosis by regulating $IP_3$ levels.
Supplementary figure 6 | Phospholipase C and calcium regulate Shiga toxin endocytosis.
Supplementary figure 7 | $IP_3$-mediated calcium release represses lipid exchange at ER/ Golgi contact sites by inhibiting OSBP recruitment and function.

**Supplementary tables**

Supplementary table 1 | Antibodies
Supplementary table 2 | Oligonucleotides

**Supplementary Data File 1** not contained in this PDF: Quantitative mass spectrometric analysis of total lipids or plasma membrane fractions from control and INPP5A-depleted HeLa cells

## Supplementary figures

Supplementary Figure 1

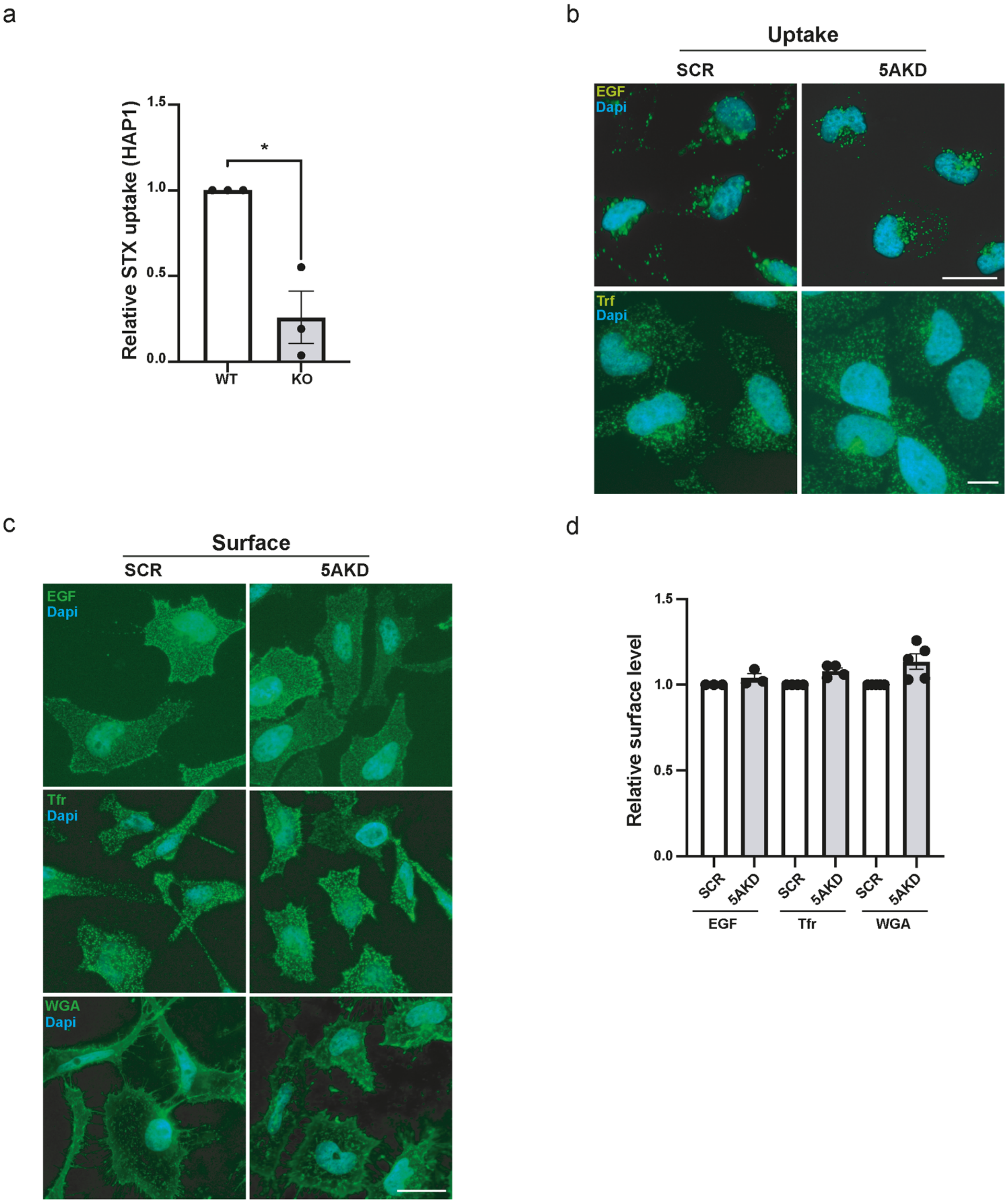


**Supplementary figure 1 | INPP5A loss impairs clathrin-independent endocytosis of bacterial Shiga toxin but not CME. (a)** Relative Shiga toxin (STX; 5 µg/ml) uptake in WT HAP-1 cells (set to 1) or *INPP5A* knockout (KO) cells. One sample t-test : p =0.0398 , t =4.862 , df =2. Data represent mean ± SEM from 3 independent experiments **(b)** Representative confocal images for fig1e. Scale bar, 25µm

(EGF) or 10µm (Tfr). **(c)** Representative confocal images of Alexa Fluor 647-labeled EGF, transferrin (Tfr), or wheat germ agglutinin (WGA) (all in green) bound to the surface of HeLa cells kept at 4°C and treated with scrambled (SCR) control siRNA or siRNA against INPP5A (5AKD). Blue, DAPI-stained nuclei. Scale bar, 25µm. **(d)** Quantification of representative data shown in Fig. S1c (SCR of each pair is set to 1) one sample t-test. EGF: $p = 0.2529$, $t = 1.589$, $df = 2$. Tf: $p = 0.0205$, $t = 4.496$, $df = 3$. WGA: $p = 0.0385$, $t = 3.037$, $df = 4$. Data represent mean ± SEM from 3 independent experiments for EGF, n=4 for Tfr and n=5 for WGA. Numerical source data are reported in the Source Data file.

Supplementary Figure 2

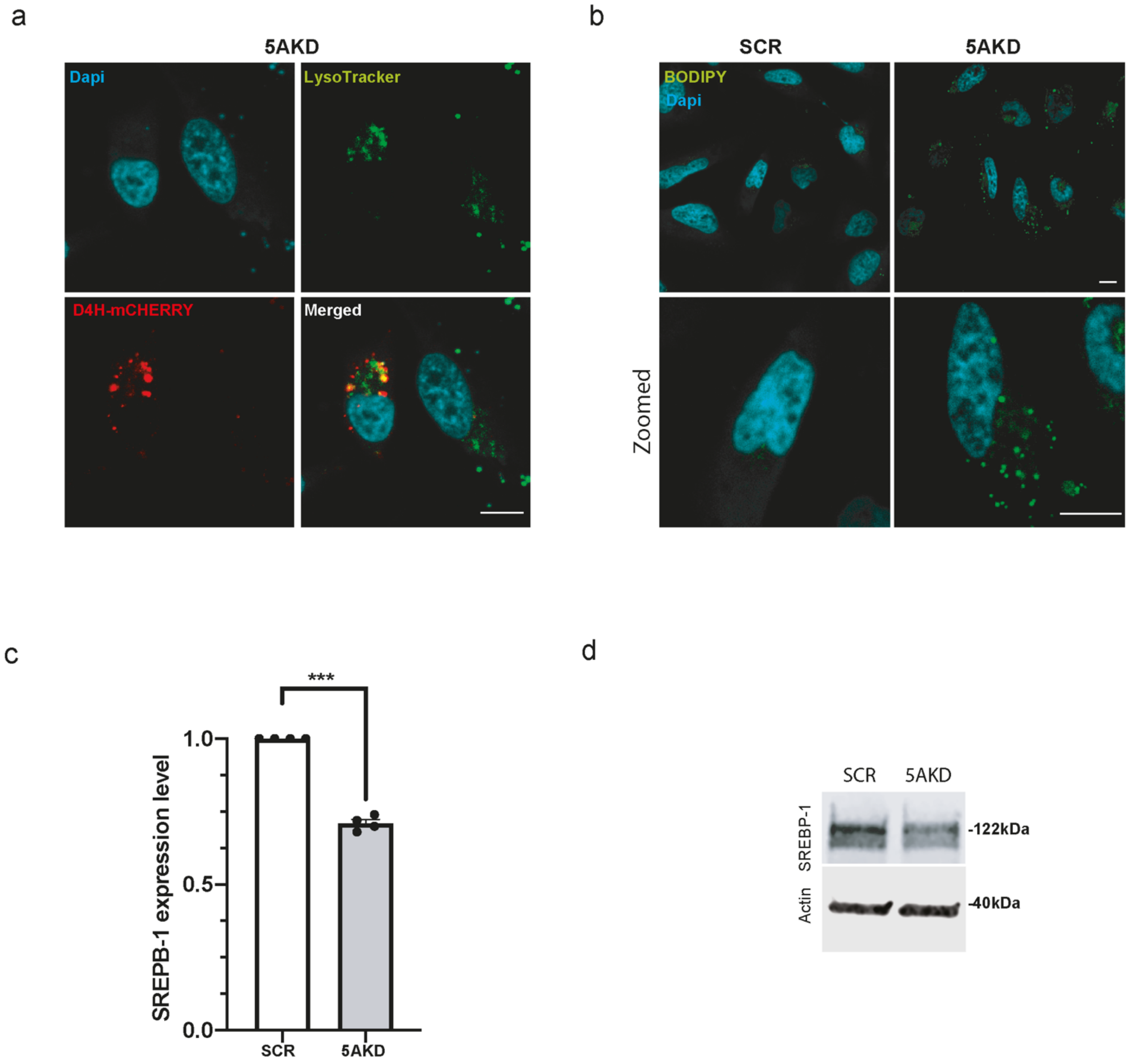


**Supplementary figure 2 | In the absence of INPP5A cholesterol is directed towards lysosomes and lipid droplets.** **(a)** Representative images of HeLa cells treated with INPP5A siRNA (5AKD), transfected with a plasmid encoding D4H-mCherry (red) to detect intracellular cholesterol, and incubated with Lysotracker (1:1000 dilution) for 60 minutes before fixation to detect lysosomes (green). Blue, DAPI-stained nuclei. Scale bar, 10 µm. **(b)** Top, low magnification views of HeLa cells treated with control (SCR) or INPP5A siRNA (5AKD) and incubated before fixation for 15 min with 2 µM of BODIPY 493/503 (4,4-Difluoro-1,3,5,7,8-Pentamethyl-4- Bora-3a,4a-Diaza-s-Indacene) (green). Bottom, zoomed images. Scale bar, 10 µm. **(c)** Quantification of representative data shown in S2d. One sample t test P=0.0002, t= 22.46 df =3. Data represent mean ± SEM from 4 independent experiments **(d)** Representative immunoblot analysis of HeLa cells treated with control (SCR) or INPP5A siRNA (5AKD) for 48 hours. Immunoblots were decorated with antibodies against SREBP-1 and β-actin as a loading control Numerical source data and unprocessed blots are reported in the Source Data file.

Supplementary Figure 3

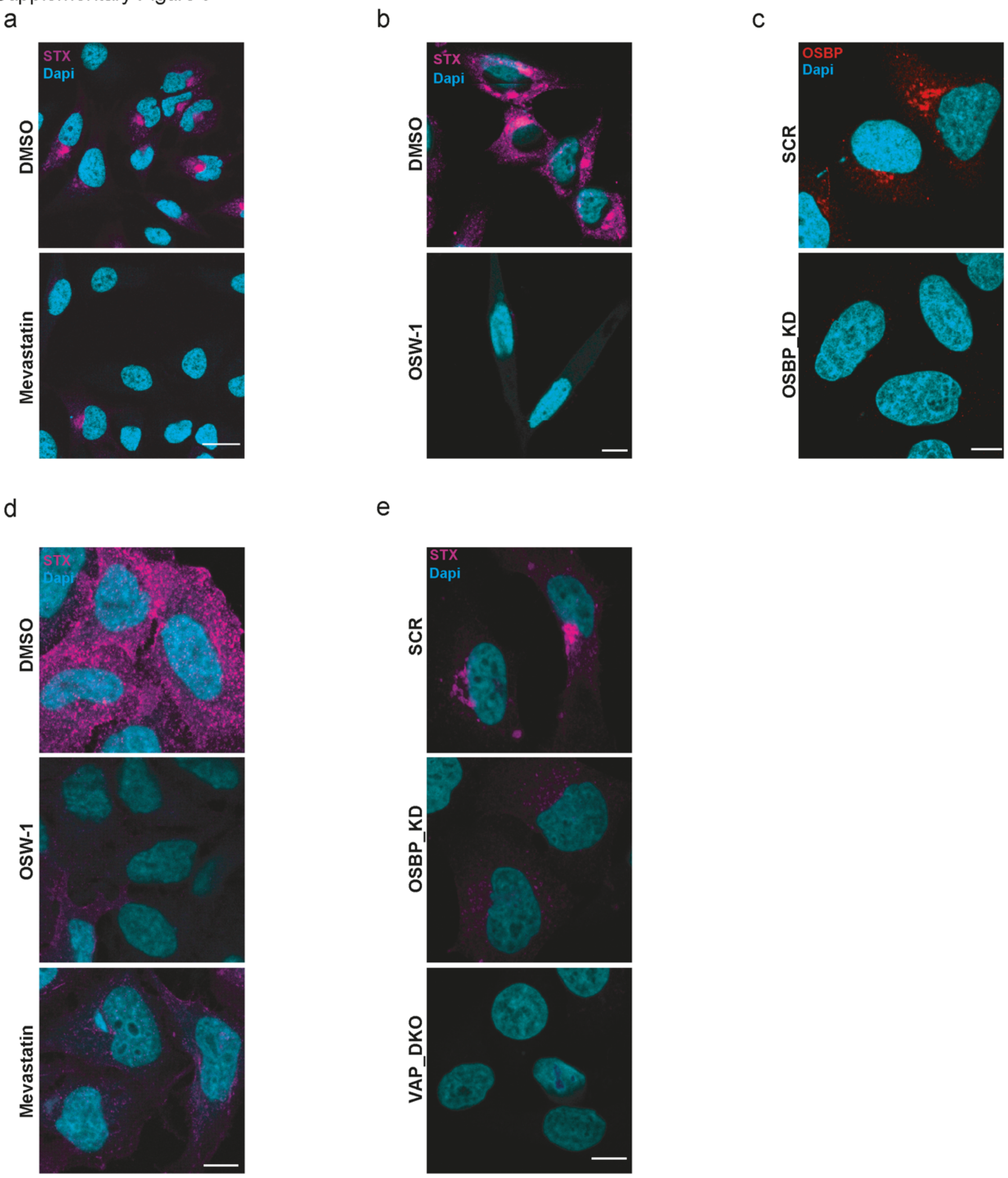


**Supplementary figure 3 | Cholesterol depletion or loss of VAP or OSBP proteins impair CIE of Shiga toxin. (a)** Representative confocal images of HeLa cells treated with control DMSO or Mevastatin (250 nM) for 24 h in presence of Serum, and incubated with labeled STX (magenta). Blue, DAPI-stained nuclei. Scale bar, 25 µm. **(b)** Representative confocal images for Fig.5g. Shiga toxin, STX (magenta) Blue, DAPI-stained nuclei. Scale Bar, 10 µm. **(c)** Representative confocal images of HeLa cells treated with control siRNA (SCR) or siRNA against OSBP (OSBP_KD) and stained for endogenous OSBP (red). Blue, DAPI-stained nuclei. Scale bar, 10 µm. **(d)** Representative confocal images for Fig.5a, Fig.5h. Shiga toxin, STX (magenta) Blue, DAPI-stained nuclei. Scale Bar, 10 µm. **(e)** Representative confocal images for Fig.5f and Fig.5i. Shiga toxin, STX (magenta) Blue, DAPI-stained nuclei. Scale Bar, 10 µm. All data are representative of data from 3 independent experiments.

Supplementary Figure 4

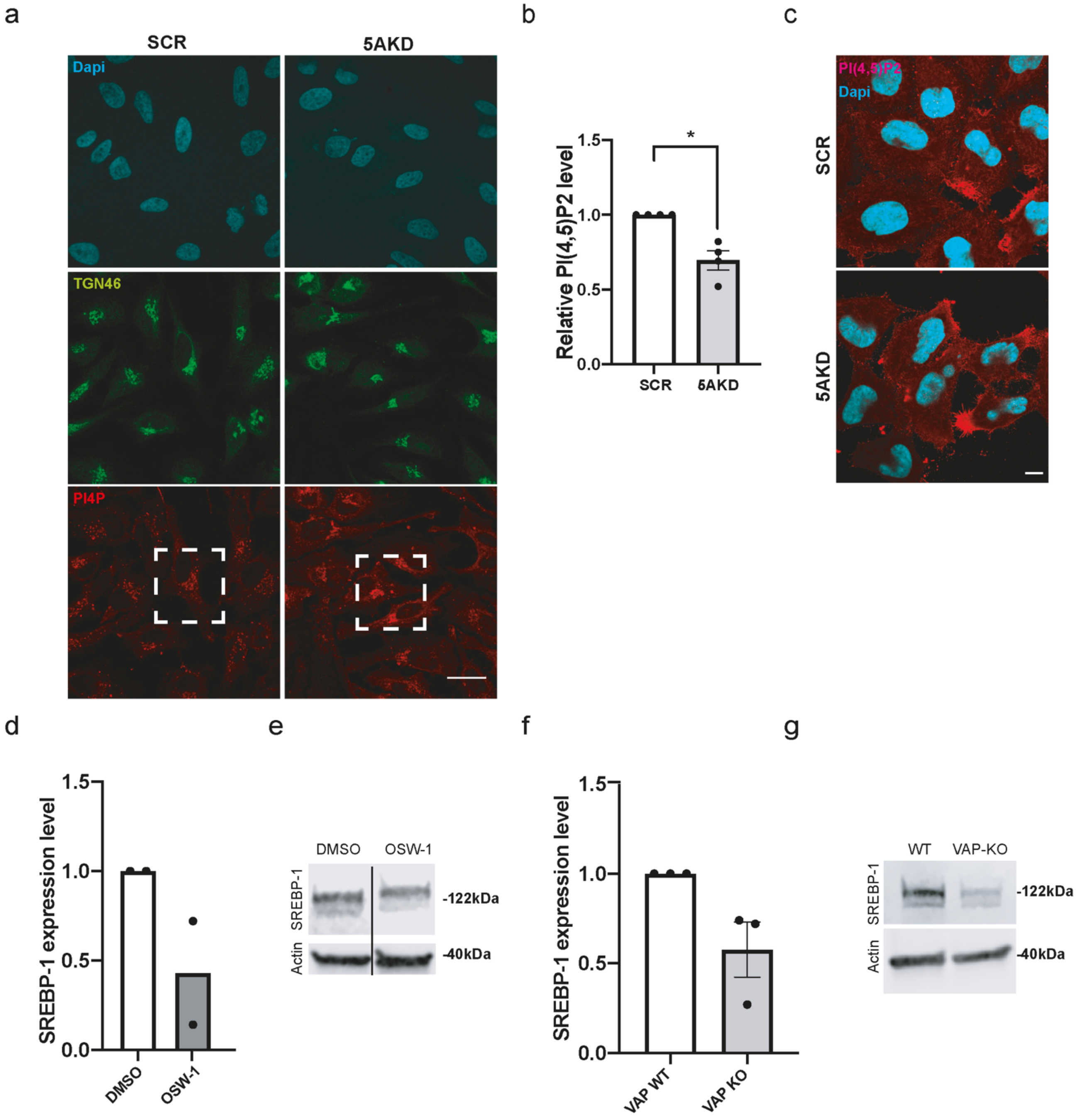


**Supplementary figure 4 | Levels of PI(4,5)P$_2$ and Golgi PI(4)P in INPP5A-depleted cells and effects of VAP or OSBP loss-of-function on SREBP-1 protein expression.** **(a)** Low magnification views representative from 5 independent experiments of HeLa treated with control siRNA (SCR) or against INPP5A (5AKD) and stained with antibodies against TGN46 (green) and PI(4)P (red). White quadrants delimit the area shown in Figure 5e. Scale bar, 25 µm. **(b)** Relative phosphatidylinositol 4,5-biphosphate [PI(4,5)P$_2$] levels in HeLa cells treated with control (SCR, set to 1) or INPP5A siRNA (5AKD) one sample t-test. p =0.0176, t =4.758, df =3. Data represent mean ± SEM from 4 independent experiments **(c)** Representative confocal images of HeLa cells treated with control (SCR) or INPP5A siRNA (5AKD) and stained for PI(4,5)P$_2$ (red). Blue, DAPI-stained nuclei. Scale Bar, 10 µm. **(d,e)** Quantification (e)

and representative immunoblot (e) analysis of HeLa cells treated with DMSO or the OSBP inhibitor OSW-1 (20 nM, 16h). Immunoblots were decorated with antibodies against SREBP-1 and β-actin as a loading control. Data represent mean ± SEM from 2 independent experiments, black vertical line indicates the fusion line of two cropped blots **(f,g)** Quantification (f) and representative immunoblot (g) analysis of wild-type (*VAP*-WT) or *VAP-A/ VAP-B* double knockout (*VAP*-DKO) HeLa cells. Immunoblots were decorated with antibodies against SREBP-1 and β-actin as a loading control. One sample t test. p=0.1101 t=2.759 df=2. Data represent mean ± SEM from 3 independent experiments. Numerical source data and unprocessed blots are reported in the Source Data file.

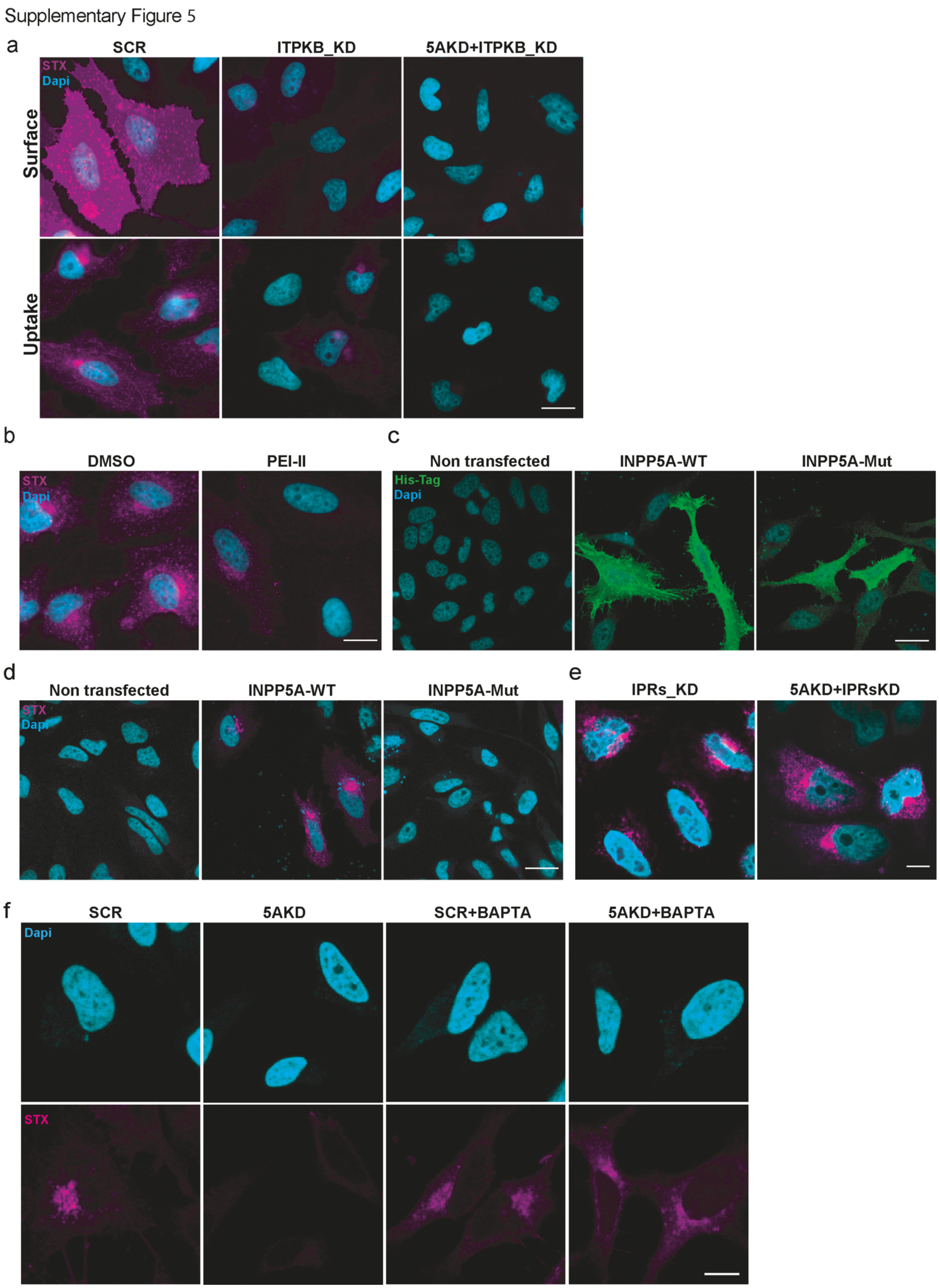


**Supplementary figure 5 | INPP5A controls Shiga toxin endocytosis by regulating IP$_3$ levels. (a)** Representative confocal images from at least 3 independent experiments for Fig.6c. Shiga toxin (STX,

magenta); blue, DAPI-stained nuclei. Scale bar, 25 µm. **(b)** Representative confocal images from 4 independent experiments for Fig. 6d Shiga toxin (STX, magenta); blue, DAPI-stained nuclei. Scale bar, 25 µm. **(c)** Representative confocal images from at least 3 independent experiments of INPP5A-depleted HeLa cells expressing WT INPP5A-myc-6xHis (INPP5A- WT) or phosphatase-inactive (INPP5A-Mut) stained with antibodies against the 6xHis-Tag (green). Blue, DAPI-stained nuclei. Scale bar, 25 µm. **(d)** Representative confocal images from at least 3 independent experiments for Fig. 6e. STX (magenta) Blue, DAPI- stained nuclei. Scale bar, 25µm. **(e)** Representative confocal images from 6 independent experiments for Fig. 6i. STX (magenta) Blue, DAPI-stained nuclei. Scale bar, 10µm. (**f)** Representative confocal images from 3 independent experiments of Figure 6h. STX (magenta) Blue, DAPI-stained nuclei. Scale bar, 10µm. Numerical source data are reported in the Source Data file.

Supplementary Figure 6

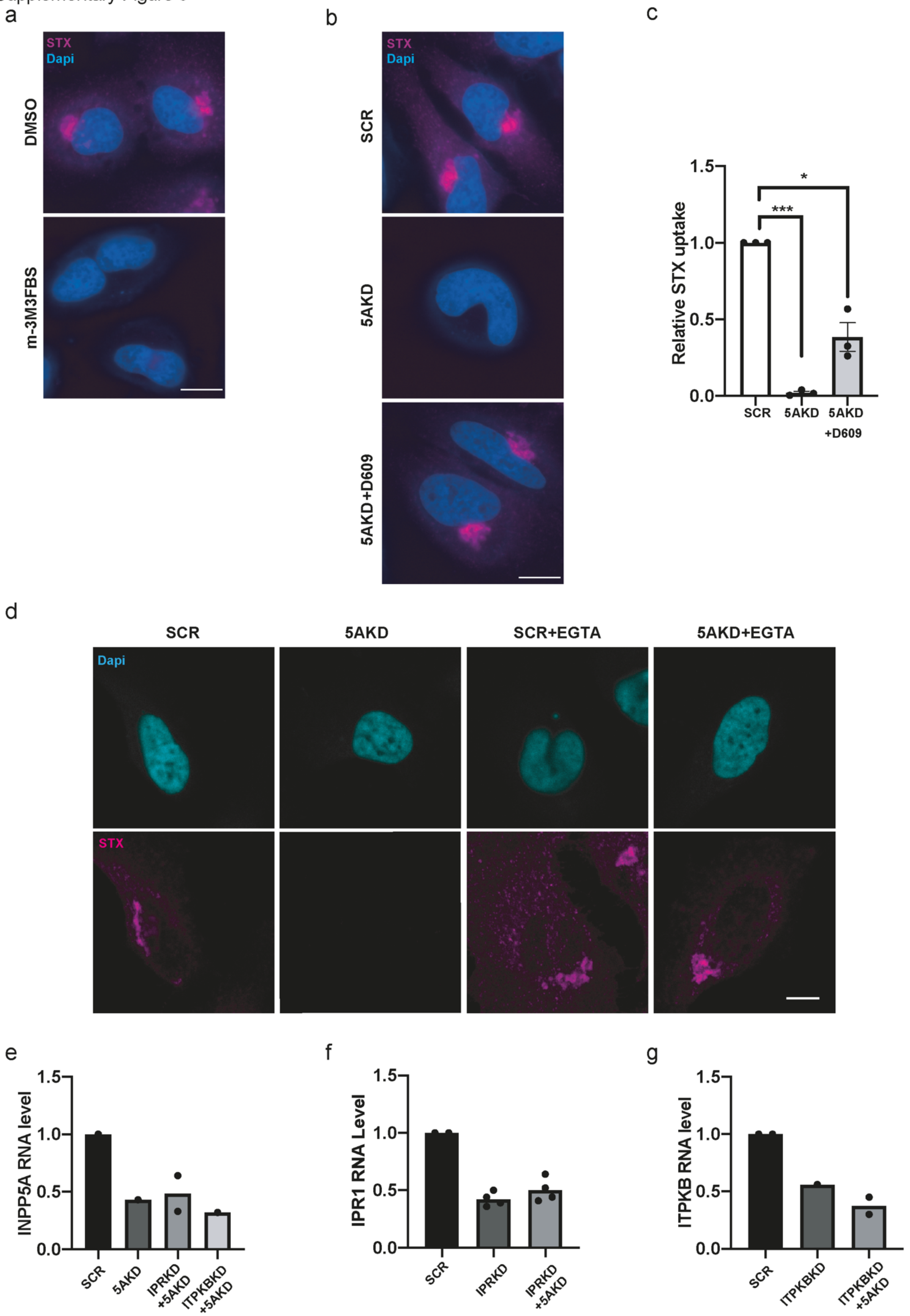


**Supplementary figure 6 | Phospholipase C and calcium regulate Shiga toxin endocytosis. (a)** Representative images for Fig.6j. Shiga toxin (STX), magenta; blue, DAPI-stained nuclei. Scale Bar,

10µm. **(b)** Representative images for Fig. S6c. Shiga toxin (STX, magenta); blue, DAPI-stained nuclei. Scale bar, 10µm. **(c)** Pharmacological inhibition of PLC activity partly rescues defective Shiga toxin endocytosis caused by loss of INPP5A. Data for SCR-siRNA transfected control cells were set to 1. One sample t-test 5AKD: p =0.0001, t =95.94, df =2. 5AKD+D609 p=0.0222 t= 6.592, df=2. Data represent mean ± SEM from three independent experiments **(d)** Defective STX endocytosis is rescued by using the cell-permeant Ca2+ chelator EGTA-AM (1 µM). Representative images where STX (magenta) Blue, DAPI-stained nuclei. Scale bar, 10 µm. **(e-f)** Efficiency of siRNA-mediated knock-downs in HeLa cells treated with scrambled control siRNA control (SCR) or specific siRNAs targeting *INPP5A* (5AKD), *IP$_3$Rs* (IPRKD), or *ITPKB* (ITPKBKD). mRNA levels were measured by real-time qPCR using specific primers for the indicated genes. (e) *INPP5A* mRNA levels. Data are representative of one (for 5AKD, ITPKKD+5AKD) or two (IPRKD + 5AKD) experiments. (f) *IP$_3$R1* mRNA levels. Data represent two (SCR) or four (IPRKD; IPRKD +5AKD) independent experiments. (g) *ITPKB* mRNA levels. Data represent one (ITPKBKD) or two (SCR; ITPKBKD +5AKD) independent experiments. Numerical source data are reported in the Source Data file.

Supplementary Figure 7

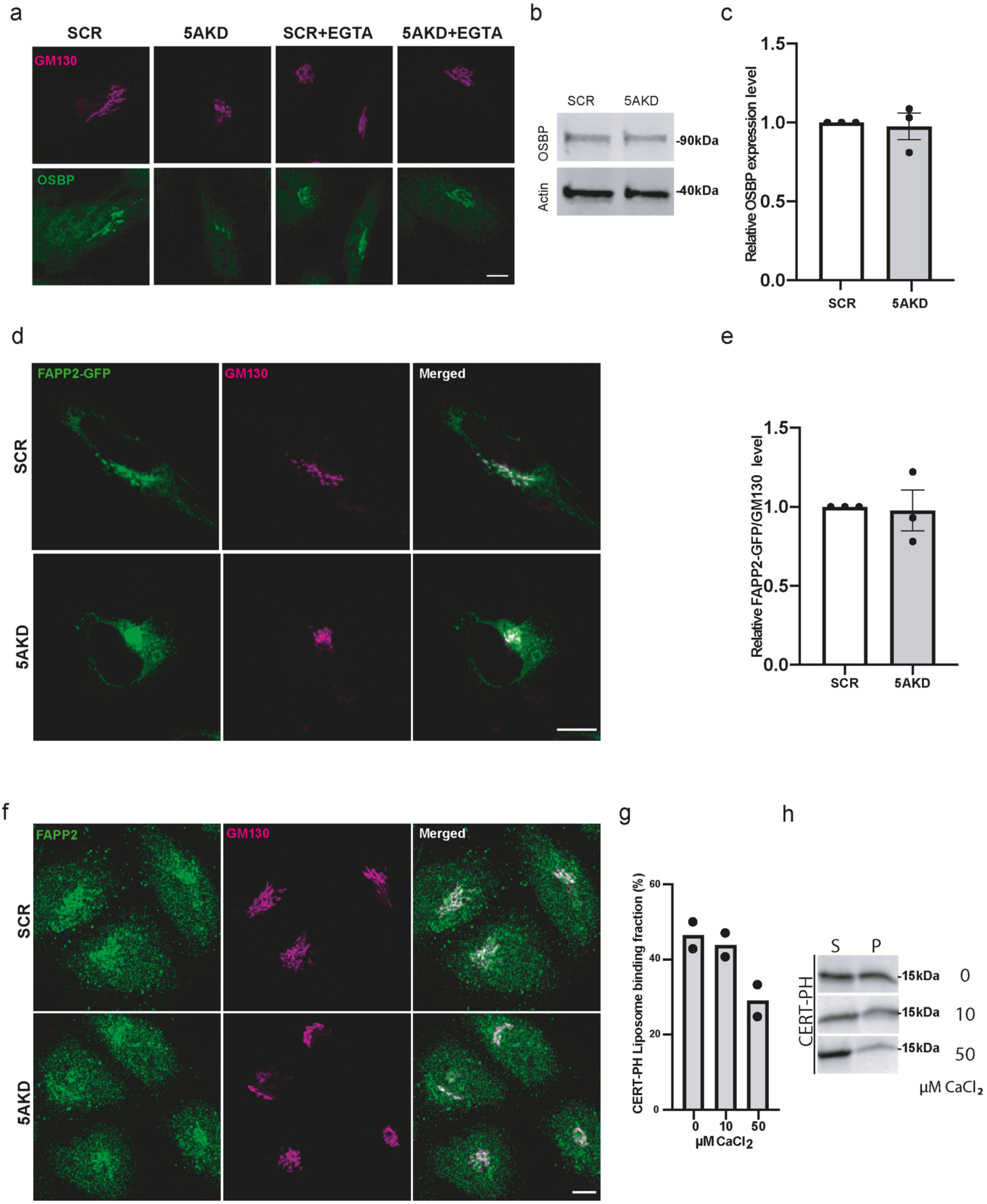


**Supplementary figure 7 | IP$_3$-mediated calcium release represses lipid exchange at ER/ Golgi contact sites by inhibiting OSBP recruitment and function. (a)** Defective OSBP localisation is rescued by using the cell-permeant Ca2+ chelator EGTA-AM (1μM). Representative images from 3 independent experiments where OSBP (Green), GM130 (Magenta) Blue, DAPI-stained nuclei. Scale

bar, 10 µm. **(b)** Representative immunoblot analysis of HeLa cells treated with control (SCR) or INPP5A siRNA (5AKD). Immunoblots were decorated with antibodies against OSBP and β-actin as a loading control. **(c)** Quantification of data shown in panel b. p=0.8015 t=0.2865 df=2 Data represent mean ± SEM from 3 independent experiments **(d)** Representative confocal images of HeLa cells treated with control (SCR) or INPP5A siRNA (5AKD) transfected with FAPP2-PH-GFP (green) for 48 hours and stained with GM130 (Magenta). Scale Bar, 10 µm. **(e)** Quantification of FAPP2-GFP colocalising with GM130 shown in Fig S7d. One sample t test p=0.8733, t=01807, df=2. Data represent mean ± SEM from 3 independent experiments **(f)** Representative confocal images of HeLa cells treated with control (SCR) or INPP5A siRNA (5AKD) stained with antibody against endogenous FAPP2 (green) and GM130 (Magenta). (Scale Bar, 10 µm). **(g)** Quantification of representative PI(4)P-liposome binding assays shown in (h). Data represent mean ± SEM from 2 independent experiments **(h)** Representative data from 3 independent experiments for Calcium effect using Liposome co-sedimentation assays to determine binding of purified recombinant CERT-PH to PI(4)P-containing liposomes. S, supernatant; P, liposomal pellet. Samples were analysed by SDS-PAGE and staining with Coomassie Blue.

## Supplementary table 1 | Antibodies

| Antibody | Species | Dilution | | Source | Catalogue number |
|---|---|---|---|---|---|
| | | ICC | IB | | |
| **Primary antibodies** | | | | | |
| GFP | ms | | 1:2500 | Clontech | 632381 |
| GM130 | ms | 1:500 | | BD transduction | 610822 |
| TGN46 | rb | 1:200 | | Abcam | ab50595 |
| INPP5A | rb | | 1:500 | ProteinTech | 21723-1-AP |
| Ceramide | ms | 1:10 | | Enzo | ALX-804-196 |
| OSBP | rb | 1:500 | | Sigma-aldrich | HPA039227 |
| Actin | ms | | 1:1000 | Sigma-aldrich | A5441 |
| SREBP1 | ms | | 5 µg/ml | Abcam | (ab3259) |
| FAPP2 | rb | 1:250 | | Gift from Antonella de matteis | N/A |
| HA-Tag | ms | | 1:1000 | Abcam | ab130275 |
| HIS-Tag | ms | 1:200 | | Novagen | 70796-3 |
| PI4P | ms | 1:100 | | Echlon | z-p004 |
| PI(4,5)P2 | ms | 1:100 | | Echlon | Z-A045 |
| **Secondary antibodies** | | | | | |
| α-ms IgG (H+L) AF488 | gt | 1:400 | | Thermo Fisher | A11029 |
| α-rb IgG (H+L) AF488 | gt | 1:400 | | | A11034 |
| α-rb IgG (H+L) AF647 | gt | 1:400 | | | A21244 |
| α-r IgG (H+L) AF488 | dk | 1:400 | | | A21208 |
| α-ms IgM (H+L) AF568 | gt | 1:400 | | | A21043 |
| α-rb IRDye680RD IgG(H+L) | gt | | 1:10000 | LI-COR Biosciences | 926 -68071 |
| α-rb IRDye800RD IgG(H+L) | gt | | 1:10000 | | 926 -32211 |
| α-msIRDye680RD IgG(H+L) | gt | | 1:10000 | | 925 -68070 |
| α-msIRDye800RDIgG(H+L) | gt | | 1:10000 | | 926 -32210 |

**Supplementary table 2 | Oligonucleotides**

| siRNA name | Sequence | Souce | Reference |
|---|---|---|---|
| Universal Negative Control #1 | n.a | Sigma MISSION | SIC001-10NMOL |
| INPP5A#1 | 5'-GCACCGCGCUCUUGGAGUU-3' | Sigma MISSION | Customized |
| OSBP | n.a | Dharmacon | L-009747-00-0020 |
| ITPKB | n.a | Dharmacon | L-006743-00-0005 |
| ITPR1 | n.a | Dharmacon | L-006207-02-0005 |
| ITPR2 | n.a | Dharmacon | L-006208-02-0005 |
| ITPR3 | n.a | Dharmacon | L-006209-02-0005 |
| PIP5K1B | n.a | Dharmacon | 8395 |
| | | | |

**Primers**

| Name | Sequence from 5´ to 3' |
|---|---|
| ITPR1-Fw | GCGGAGGGATCGACAAATGG |
| ITPR1-Rev | TGGGACATAGCTTAAAGAGGCA |
| ITPKB-Fw | TCTCCTCATCCTACGAAGACTCA |
| ITPKB-Rev | GCTCACTCTAGGTTTCTGCTGG |
| INPP5A-Fw | ACACGAACATGGCACTAGGA |
| INPP5A-Rev | GGGCGTGCTCTCTAAGGTAT |
