# Supplementary material for: Inositol triphosphate-triggered calcium release blocks lipid exchange at endoplasmic reticulum-Golgi contact sites
Source: Nat Commun. 2021 May 11;12:2673. doi: 10.1038/s41467-021-22882-x (PMC8113574; doi:10.1038/s41467-021-22882-x)
Supplement: Supplementary file 2 — Description of Additional Supplementary Files [file 41467_2021_22882_MOESM2_ESM.pdf]

Description of Additional Supplementary Files

Supplementary Data 1: Quantitative mass spectrometric analysis of total lipids or plasma membrane fractions from control and INPP5A-depleted HeLa cells
