# Supplementary material for: Inositol triphosphate-triggered calcium release blocks lipid exchange at endoplasmic reticulum-Golgi contact sites
Source: Nat Commun. 2021 May 11;12:2673. doi: 10.1038/s41467-021-22882-x (PMC8113574; doi:10.1038/s41467-021-22882-x)
Supplement: Supplementary file 4 — Reporting Summary [file 41467_2021_22882_MOESM4_ESM.pdf]

nature research

Corresponding author(s): Volker Haucke

Last updated by author(s): Apr 2, 2021

Reporting Summary

Nature Research wishes to improve the reproducibility of the work that we publish. This form provides structure for consistency and transparency in reporting. For further information on Nature Research policies, see our Editorial Policies and the Editorial Policy Checklist.

Statistics

For all statistical analyses, confirm that the following items are present in the figure legend, table legend, main text, or Methods section.

n/a	Confirmed	
☐	☒	The exact sample size (*n*) for each experimental group/condition, given as a discrete number and unit of measurement
☐	☒	A statement on whether measurements were taken from distinct samples or whether the same sample was measured repeatedly
☐	☒	The statistical test(s) used AND whether they are one- or two-sided<br>*Only common tests should be described solely by name; describe more complex techniques in the Methods section.*
☒	☐	A description of all covariates tested
☐	☒	A description of any assumptions or corrections, such as tests of normality and adjustment for multiple comparisons
☐	☒	A full description of the statistical parameters including central tendency (e.g. means) or other basic estimates (e.g. regression coefficient) AND variation (e.g. standard deviation) or associated estimates of uncertainty (e.g. confidence intervals)
☐	☒	For null hypothesis testing, the test statistic (e.g. *F*, *t*, *r*) with confidence intervals, effect sizes, degrees of freedom and *P* value noted<br>*Give P values as exact values whenever suitable.*
☒	☐	For Bayesian analysis, information on the choice of priors and Markov chain Monte Carlo settings
☒	☐	For hierarchical and complex designs, identification of the appropriate level for tests and full reporting of outcomes
☒	☐	Estimates of effect sizes (e.g. Cohen's *d*, Pearson's *r*), indicating how they were calculated

Our web collection on statistics for biologists contains articles on many of the points above.

Software and code

Policy information about availability of computer code

Data collection	Data for immunofluorescence images was acquired using FIJI Version : 2.1.0/1.53c<br>Quantitative values from Western Blots were acquires using the Licore Odyssey Software for Quantitative Western Blotting Version:Fc<br>Quantifications of SDS bands from the Liposomes Binding Assay were performed imgaeLab 6.0.1 Biorad.<br>Microsoft Excel (version 16.46) for data storage and processing.<br>Numbers for MAC version 10.0
Data analysis	All statistical tests were performed using Graphpad Prism. Version : PRISM8 for MAC 8.4.3(471)

For manuscripts utilizing custom algorithms or software that are central to the research but not yet described in published literature, software must be made available to editors and reviewers. We strongly encourage code deposition in a community repository (e.g. GitHub). See the Nature Research guidelines for submitting code & software for further information.

Data

Policy information about availability of data

All manuscripts must include a data availability statement. This statement should provide the following information, where applicable:

- Accession codes, unique identifiers, or web links for publicly available datasets
- A list of figures that have associated raw data
- A description of any restrictions on data availability

All lipidomics data are reported in Supplementary Data File 1 and have been deposited to the Metabolights database where they are available under accession number MTBLS2444. All other data are available in the main article or the Supplementary Information files.
Numerical source data for Figs. 1–7 and uncropped versions of blots and gels are provided in the Source Data File.

Field-specific reporting

Please select the one below that is the best fit for your research. If you are not sure, read the appropriate sections before making your selection.

- [x] Life sciences
- [ ] Behavioural & social sciences
- [ ] Ecological, evolutionary & environmental sciences

For a reference copy of the document with all sections, see nature.com/documents/nr-reporting-summary-flat.pdf

Life sciences study design

All studies must disclose on these points even when the disclosure is negative.

Sample size	Sample sizes were not chosen based on pre-specified effect size but selected based on commonly adopted standards in the field, resulting in statistically meaningful comparison , Multiple independent experiements were carried out using several sample replicates as detailed in the figure legends and Data reproducibility section within methods.
Data exclusions	No samples were excluded from analysis.
Replication	All experiments were carried out under standard and clearly defined conditions, and were replicated successfully by at least one researcher and all attempts of replication were successful. The number of replicates of each experiment is specified in the corresponding figure legend and data and reproducibility section within the Methods.
Randomization	No animals have been used for this study, and No randomization was needed for the experiment with cultured cell line as cells were passaged in the same step from one parental cell dish for all groups in each experiment.
Blinding	Immunofluorescence images were captured blindly by selecting cells in the DAPI channel (when available) or the GM130 channel when OSBP localisation was monitored. Cells for immunoblots and lipidomics, or samples for liposome binding assays were not collected or processed blindly since knowledge of the characteristic of each sample is necessary for data generation.<br>For analysis the person who did the analysis was the experimenter, so he/she knew the conditions that were analysed.

Reporting for specific materials, systems and methods

We require information from authors about some types of materials, experimental systems and methods used in many studies. Here, indicate whether each material, system or method listed is relevant to your study. If you are not sure if a list item applies to your research, read the appropriate section before selecting a response.

Materials & experimental systems

n/a	Involved in the study	
☐	☒	Antibodies
☐	☒	Eukaryotic cell lines
☒	☐	Palaeontology and archaeology
☒	☐	Animals and other organisms
☒	☐	Human research participants
☒	☐	Clinical data
☒	☐	Dual use research of concern

Methods

n/a	Involved in the study	
☒	☐	ChIP-seq
☒	☐	Flow cytometry
☒	☐	MRI-based neuroimaging

Antibodies

Antibodies used	All information regarding antibodies (supplier name, catalog number, application, dilution etc). are provided in a separate table (Table 1)<br>Primary antibodies :

GFP: species mouse, dilution : 1:2500, Clontech Cat# 632381
GM130: species mouse, dilution 1:500, BD transduction Cat#610822
TGN46 species rabbit, dilution 1:200, Abcam Cat#ab50595
INPP5A species rabbit, dilution 1:500, ProteinTech Cat#21723-1-AP
Ceramide species mouse , dilution 1:10, Enzo Cat#ALX-804-196
OSBP species rabbit , dilution 1:500, Sigma-aldrich Cat#HPA039227
Actin species mouse , dilution 1:1000, Sigma-aldrich Cat#A5441
SREBP1 species mouse, dilution 5 µg/ml, Abcam Cat#(ab3259)
FAPP2 species mouse, dilution 1:250, Gift from Antonella de matteis N/A (NOT commercialized)
HA-Tag species mouse, dilution 1:1000, Abcam Cat#ab130275
HIS-Tag species mouse , dilution 1:200, Novagen Cat#70796-3
PI4P species mouse, dilution 1:100, Echlon Cat#z-p004
PI(4,5)P2 species mouse, dilution 1:100, EchlonCat# Z-A045
Secondary antibodies :
Goat anti mouse IgG (H+L) AF488, dilution 1:400 Thermo Fisher Cat#A11029
Goat anti rabbit IgG (H+L) AF488, dilution 1:400 Thermo Fisher Cat#A11034
Goat anti rabbit IgG (H+L) AF647, dilution 1:400 Thermo Fisher Cat#A21244
Donkey anti rabbit IgG (H+L) AF488, dilution 1:400 Thermo Fisher Cat#A21208
Goat anti mouse IgM (H+L) AF568, dilution 1:400 Thermo Fisher Cat#A21043
Goat anti rabbit IRDye680RD IgG(H+L), dilution 1:10000 LI-COR Biosciences Cat#926 -68071
Goat anti rabbit IRDye800RD IgG(H+L), dilution 1:10000 LI-COR Biosciences Cat#926 -32211
Goat anti mouse IRDye680RD IgG(H+L), dilution 1:10000 LI-COR Biosciences Cat#925 -68070
Goat anti mouse IRDye800RDIgG(H+L), dilution 1:10000 LI-COR Biosciences Cat#926 -32210

Validation

INPP5A specific antibodies were validated in Human cells by immunoblotting following siRNA-mediated knockdown of the target protein or using INPP5A-Knocked-Out Hap-1 cells lysates. OSBP specific antibody was validated by immunofluoresence in human cells following siRNA-mediated knockdown of the target protein and checking its staining pattern according to what has been published. All other antibodies used for immunoblotting were validated by including appropriate molecular weight markers and determining if the protein band had the expected molecular weight. For antibodies used for immunofluorescence, antibody specificity was tested by adding positive and negative controls and checking their staining patterns according to scientific literature.

Eukaryotic cell lines

Policy information about cell lines

Cell line source(s)

HEK293T, Cos7, HeLa cells were obtained from ATCC. HAP1 cells were obtained from Horizondiscovery. VAP-A/B double KO or parental HeLa cells have been described before (Cell. 2016 Jul 14; 166(2): 408–423 referred to in the main manuscript) and were a kind gift from the laboratory of Pietro De Camilli (Yale Univ. School of Medicine, New Haven, CT, USA).

Authentication

Cell lines from ATCC are regularly authenticated by STR profiling and were used by us without further authentication. Cell lines obtained from HorizonDiscovery were checked for their INPP5A expression (RTPCR). Other sources were not authenticated by us.

Mycoplasma contamination

Cell lines were regularly tested for mycoplasm contamination and were not contaminated

Commonly misidentified lines
(See ICLAC register)

No commonly misidentified cell lines were used in the study.
